# Precision of predictive nomograms for lymph node metastasis of thyroid cancer from Chinese real-world study: a systematic review and meta-analysis

**DOI:** 10.3389/fendo.2025.1617563

**Published:** 2025-07-09

**Authors:** Yongke Wu, Yuanhao Su, Yiyuan Zhao, Nassuf Mourdi, Zhidong Wang

**Affiliations:** Department of Geriatric General Surgery, The Second Affiliated Hospital, Xi’an Jiaotong University, Xi’an, Shaanxi, China

**Keywords:** papillary thyroid carcinoma, lymph node metastasis, thyroidectomy, nomogram, meta-analysis, China

## Abstract

**Background:**

Current guidelines lack nomograms to predict lymph node metastasis (LNM) in thyroid carcinoma (TC) in China. Nomograms are simple, accurate tools to estimate the probability of specific events and have been extensively developed to predict LNM in TC. However, few effective nomograms have been validated in clinical practice.

**Methods:**

The recommendations of the Cochrane Prognosis Methods Group were implemented in this systematic review. We conducted searches in PubMed, Web of Science, and Scopus for published research. The nomogram was categorized based on outcomes. We summarized the key characteristics and effectiveness of the nomogram and assessed the overall risk of bias (ROB). We employed random-effects and bivariate mixed-effects models to estimate the efficacy of the nomogram group and its predictive reliability.

**Results:**

The systematic review identified 57 nomogram models from China, of which only 14 had external validation cohorts. While the applicability was acceptable, the heterogeneity among the included nomograms was substantial, leading to a high overall risk of bias (ROB). Ultrasound information was utilized in nearly all studies. Size, extrathyroidal extension (ETE), tumor consistency index (TCI), and multifocality are commonly employed independent risk factors. Both outcome models showed good to excellent predictive efficacy. However, the performance of models that integrate radiomics with clinical features was inferior to those using ultrasound alone.

**Conclusions:**

The feature-combined model offers several potential outcomes and advantages for clinical practice in China. Additionally, the systematic review serves as a reference tool for physicians to select appropriate nomograms based on individual clinical needs. Future research should focus on external validation and evaluation to minimize limitations in clinical utility.

## 1 Introduction

A long-term study showed that the incidence of thyroid cancer (TC) has rapidly increased in recent decades (1). According to the latest Chinese cancer statistics, there were 466,100 new TC cases, and the incidence of TC was the third most prevalent in 2022 (2). Differentiated thyroid cancer (DTC), which includes papillary thyroid carcinoma (PTC), accounts for more than 80% of all thyroid cancers (3, 4). Although PTC generally has low-grade malignancy and a favorable long-term prognosis, the recurrence rate is relatively high, up to 20%, and lymph node metastasis (LNM) is about 30%–90%, primarily in the central compartment of the neck (5).

Current clinical guidelines differ in diagnosing and treating LNM from PTC. In China, individualized management of low-risk patients at stage cN0 (clinically uninvolved central neck lymph nodes) without associated high-risk factors (6) is emphasized, while the American Joint Committee on Cancer (AJCC) staging places more focus on patient recurrence and survival (7, 8). The National Comprehensive Cancer Network (NCCN) recommends considering prophylactic central neck dissection (pCND) for patients with cN0 (9, 10). However, the NCCN guidelines overrule previous conclusions based on the results of only one study (11). The American Thyroid Association (ATA) guidelines suggest pCND for patients with cN0 PTC, particularly for tumors >4 cm or with extrathyroidal extensions (ETE) (12). Yet, relying solely on simple imaging features as risk factors may not sufficiently predict many subclinical LNM of TC. Prophylactic neck dissection (pND) facilitates more complete staging of the cervical lymph nodes and could enhance prognosis while guiding subsequent treatment and follow-up management. However, patients must consider the risk of over-diagnosis and over-treatment (13). Highly accurate detection of LNM from PTC is a crucial factor influencing treatment outcomes for patients.

The main tests for detecting LNM in TC are ultrasound, fine needle aspiration (FNA), and computed tomography (CT). Ultrasound and FNA with thyroglobulin washout (FNA-Tg) significantly improve the diagnostic rate of PTC and LNM (14). However, due to non-standardization and technical limitations, they cannot be used as the only standard method for detecting LNM (15–18). Although CT and positron emission tomography (PET) compensate for the low sensitivity of these tests (19–21), their high cost and complexity are drawbacks. No specific laboratory indicator has been found to have a diagnostic value for clinical practice. While LNM may not be detectable on preoperative imaging or clinical examination, it may still be present or recur after thyroidectomy (22, 23).

A nomogram serves as a user-friendly tool to predict individual probabilities of specific patient events, such as tumor metastasis and prognosis, as well as to construct risk stratification, which helps to personalize medical care and counsel (24). The developed nomograms varied based on the data set characteristics and the outcome variables. Nevertheless, few predictive models have been implemented in clinical practice. The most significant drawback is the lack of adequate feature interpretation and model validation (both internal and external), making the model challenging to apply in clinical settings.

The standardization of diagnosis and treatment for TC and its LNM needs urgent resolution in China. In this article, we first conducted a systematic review and meta-analysis of multivariate nomograms for predicting LNM related to TC in a real-world Chinese study. We summarize nomograms based on clinical data, identify those suitable for further research, and provide recommendations for individual patients and reference tools for clinical practice.

## 2 Materials and methods

We conducted this systematic review according to the recommendations of the Cochrane Prognosis Methods Group (25, 26). The systematic review protocol was prospectively registered on the PROSPERO website (registration ID: CRD42024548413).

### 2.1 Eligibility criteria

We utilized the CHARMS checklist (27) and TRIPOD-SRMA checklist (28) for systematic reviews of prediction model studies and employed the PICOTS scheme to define the review questions (**Table 1**): What is the clinical value of nomograms in predicting LNM in (PTC)?

**Table 1:** PICOTS system for predictive models of LNM of papillary thyroid cancer.

P	Population	Patients with papillary thyroid cancer
I	Index model(s)	All developed predictive models for CLNM and LLNM and corresponding external validation studies
C	Comparator	Not predefined
O	Outcome(s)	CLNM or LLNM
T	Timing	Any moment of prediction at diagnosis of LNM
S	Setting	Not specified

Studies were eligible if they (I) matched the research question by including patients with PTC and (II) presented a multivariable prognostic nomogram that was based on development and validated either internally or externally. (III) The model’s accuracy and calibration for the nomograms were graphically reported. (IV) Only full-text manuscripts published in English were included. (V) Additionally, only studies involving adult TC were considered.

### 2.2 Databases searched

A systematic search strategy was conducted in PubMed, Web of Science, and Scopus using the following keywords: (thyroid cancer OR differentiated thyroid cancer OR papillary thyroid carcinoma) AND (nomogram OR nomograms) AND (CLNM OR LLNM OR central lymph node metastasis OR lateral lymph node metastasis). To gather as many detailed papers as possible, we developed a search formula by combining subject terms with free words (**Supplementary Material S1**). Based on the varying outcomes, we categorized the articles that passed the screening into two groups: CLNM-nomograms and LLNM-nomograms.

### 2.3 Data extraction process

We extracted data from the following fields: paper information, participant information, sample size, predictors, outcomes to be predicted, model performance, and information on internal and external validation. If different models are reported in the same article, we consider them to be separate independent nomograms. However, as is known from the methodological behavior of prediction models and the large number of systematic reviews reported (29, 30), calibration performed poorly and was unsuitable for further assessment. Therefore, we evaluated the inadequacy of calibration reporting. Similarly, we collected an index of model efficacy (AUC, sensitivity, and specificity). We extracted sensitivity and specificity from ROC curves for articles without descriptions using Origen2021 software and screened sensitivity and specificity using the optimal Jordon index.

### 2.4 Risk of bias assessment

The Prediction model Risk of Bias Assessment Tool (PROBAST) evaluated the quality of predictive model studies. PROBAST comprises 20 signaling questions utilized for ROB assessment across four domains: participants, predictors, outcomes, and analysis. The results of the PROBAST analysis were reported for each domain and categorized as follows: bias (low risk, high risk, unclear) and applicability (low concern, high concern, unclear). Any potential disagreements were discussed and resolved through constructive commentary and mutual consensus among all authors.

### 2.5 Statistical analysis

Different outcome nomograms were grouped due to the lack of validation studies in the meta-analysis and the heterogeneity of the nomograms. Considering the specificity of LNM models in TC, no clear model is applied to clinical practice in the guidelines. We grouped the models with the same outcome together and investigated the overall performance of the nomogram groups by pooling the C-index. The C-index is equivalent to the area under the receiver operating characteristic (ROC) curve for models with binary endpoints (31). We established cutoff values of 0.6, 0.75, and 0.85 for moderate, significant, and excellent discrimination accuracy in nomograms.

We included the C-index that best accounted for the risk of overfitting in development studies, favoring the internal validation cohort and validation through resampling by bootstrapping instead of the development cohort without validation—subgroup analysis based on modeling variables and model type (ultrasound and combined radiomic). A sensitivity, specificity, and AUC meta-analysis was conducted using bivariate mixed-effects and random-effects models. We utilized the statistical v.4.2.2 software (R Development Core Team, Vienna, http://www.R-project.org).

## 3 Results

### 3.1 Nomogram search and study characteristics

**Figure 1:**
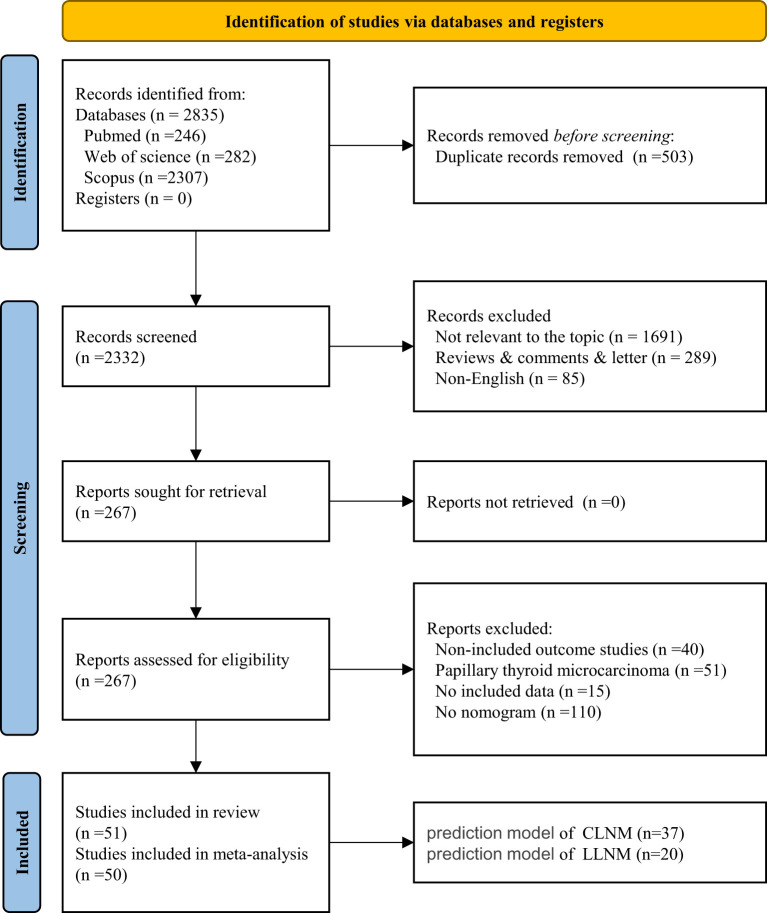
PRISMA flow chart of the study selection process.

A total of 2,835 studies were identified through the search strategy and by merging three databases. After removing duplicates, the titles and abstracts of 2,332 articles were reviewed. By carefully examining the titles and abstracts, we excluded literature that was not relevant to the topic (*n* = 1,691), along with types of reviews, comments, letters (*n* = 289), and non-English articles (*n* = 85). We conducted a full-text review of 267 articles. Ultimately, 51 studies, comprising 57 models, were included in the systematic review, while 50 studies, totaling 56 models, were included in the meta-analysis (**Figure 1**). All included studies are detailed in **Supplementary Material S2**. The heat map of China was created based on the frequency and total number of included models to illustrate the establishment and popularity of LNM models of PTC, with variations potentially linked to the local medical care level and the population’s epidemiology (**Supplementary Material S3**).

In the CLNM group, a total of 37 nomograms were included, with 25,102 patients enrolled in the training set. A total of 9,993 cases were used for internal validation, while 1,958 cases were utilized for external validation. All studies were conducted in China, spanning from 2011 to 2024. A total of 31 studies employed internal validation procedures. Specifically, 19 studies used split-sample validation with varying proportions, Tong’s study utilized multicenter internal validation (32), eight studies verified splitting samples by admission time, and the remaining two studies employed internal validation via bootstrap resampling. Five studies (including six nomogram models) performed external validation. The primary source of external validation was split samples of different proportions. The detailed literature information is shown in **Table 2**.

**Table 2:** **S**ummary of the publications included in the systematic review, highlighting paper information and nomogram prediction outcome (CLNM).

Author	year	Period	Type	Source data	Outcome	Total sample enrolled	Statistical model	Validation method	Sample size (trD)	Sample size (InV)	Sample size (EnV)
Chang, L	2023	2011.3-2018.6	D/V	RC/MC	CLNM	3,359	Logistic regression	Split-sample V (7:3) + EXV	2,114	906	339
Chang, Q	2022 (33)	2018.8-2021.11	D/V	RC	CLNM	1,324	Logistic regression	Split-sample V (3:1) + EXV	993	331	631
Chen, F	2023	2018.11-2021.12	D/V	RC	CLNM	691	Logistic regression	Bootstrap resampling	691	691	NI
Chen, H	2024	2020.1-2021.12	D/V	RC	CLNM	466	Logistic regression	Split-sample V (7:3)	326	140	NI
Chen, Q	2023	2017.1-2021.10	D/V	RC	CLNM	216	LASSO regression + logistic regression	Split-sample V (5:5)	108	108	NI
Dai, Q	2022 (34)	2019.12-2024-4	D/V	RC	CLNM	822	Logistic regression	Split-sample V (7:3)	575	247	NI
Dai, Q	2022 (34)	2019.12-2024-4	D/V	RC	CLNM	822	Logistic regression	Split-sample V (7:3)	575	247	NI
Deng, Y	2023	2017.1-2020.10	D/V	RC	CLNM	989	Logistic regression	Split-sample V (8:2)	791	198	NI
Du, J	2023	2018.1-2022.1	D/V	RC	CLNM	400	Logistic regression	Split-sample V (7:3)	280	120	NI
Feng, J	2021	2019.7-2020.6	D/V	RC	CLNM	886	Logistic regression	Split-sample V (7:3)	617	269	NI
Feng, J	2024	2022.1-2023.1	D/V	RC	CLNM	1,069	Logistic regression	Split-sample V (7:3)	748	321	NI
Gao, X	2021	2019.1-2020.7	D/V	RC	CLNM	296	Logistic regression	Randomization	296	100/95	NI
He, L	2024	2020.8-2023.4	D/V	RC	CLNM	684	Logistic regression	Split-sample by admission	495	189	NI
Hei, H	2023	2012.6-2014.12	D/V	RC	CLNM	619	Logistic regression	Split-sample by admission	436	183	NI
Hu, Q	2021	2016.1-2019.12	D	RC	CLNM	418	Logistic regression	NI	418	NI	NI
Hu, W	2023	2017.1-2021.12	D/V	RC	CLNM	133	Logistic regression + comparative incorporation	Split-sample V (7:3)	94	39	NI
Huang, C	2020	2014.7-2019.6	D/V	RC	CLNM	818	LASSO	Split-sample by admission	512	306	NI
Huang, Y	2023	2019.9-2021.5	D	RC	CLNM	344	Logistic regression	NI	344	NI	NI
Jiang, L	2023	2018.6-2020.4	D/V	RC	CLNM	211	LASSO + logistic regression	Split-sample V (7:3)	148	63	NI
Li, J	2022	2015.1-2020.3	D/V	RC	CLNM	729	(LASSO) regression and multivariate logistic regression	Split-sample V (6:4)	431	298	NI
Lin, P	2021	2016.10-2021.4	D	RC	CLNM	423	logistic regression	NI	423	NI	NI
Liu, W	2023 (35)	2011.1-2022.4	D/V	MC	CLNM	6,650	Logistic regression	Split-sample V (7:3)	4,247	1,821	582
Pang, J	2023 (36)	2020.1-2021.12	D/V	RC	CLNM	1,394	LASSO + logistic regression	Split-sample V (7:3)	976	418	NI
Qiao, D	2024	2018.9-2023.1	D/V	RC	CLNM	1,392	Logistic regression	Split-sample by admission	1,009	383	NI
Song, X	2024	2021.1-2023.2	D/V	PC	CLNM	228	Logistic regression	Split-sample by admission	128	100	NI
Sun, F	2021 (37)	2016.1-2020.6	D/V	MC	CLNM	1,585 + 406	Logistic regression	Split-sample V (7:3) + EXV	1,094	491	406
Wang, Z	2022	2018.1-2019.10	D/V	RC	CLNM	2554	logistic regression	Split-sample V (7:3)	1,787	767	NI
Wei, L	2024	2019.7-2023.4	D/V	RC	CLNM	114	Logistic regression	Split-sample v (2:1)	76	38	NI
Wen, Q	2022	2021.3-2022.3	D/V	RC	CLNM	353 + 68	Logistic regression	Split-sample by admission	353	68	NI
Xue, J	2023	2020.9-2022.12	D/V	RC	CLNM	129	LASSO + logistic regression	Split-sample v (7:3)	90	39	NI
Xue, T	2021	2016.10-2021.3	D	RC	CLNM	379	Logistic regression	NI	379	NI	NI
Yang, Z	2020 (38)	2016.6-2019.6	D/V	RC	CLNM	14,38	Logistic regression	Split-sample by admission	1,252	186	NI
Zeng, B	2022 (39)	2019.1-2021.4	D/V	RC	CLNM	747	Logistic regression	Bootstrap resampling	747	374	NI
Zhang, H	2020	2015.1-2018.4	D	RC	CLNM	214	Logistic regression	NI	214	NI	NI
Zhou, S	2020	2018.1-2018.9	D/V	RC	CLNM	609 + 326	LASSO + logistic regression	Split-sample by admission	609	326	NI
Tong, Y	2022 (32)	2019.1-2019.6	D/V	MC	CLNM	720	Logistic regression	MC	300	143	277
Zhao, D	2024	2018.2-2021.2	D/V	RC	CLNM	609	Logistic regression	Split-sample V (7:3)	426	183	NI

D, develop model; V, validate model; RC, retrospective cohort study; MC, multicenter cohort study; NI, no information.

In the LLNM group, more than 17,673 patients were enrolled in the development group, while 6,348 were used in the validation group, of which 2,103 were utilized for external validation. All studies were conducted within the Chinese population. Internal validation was applied in 15 studies. Specifically, seven studies employed split samples in varying proportions, and bootstrap validation was implemented in three studies (including four nomograms). Only one study utilized internal multicenter validation (32). Although two studies (three nomograms) included internal validation cohorts, we did not find specific instructions in the articles (40, 41). Eight studies conducted external validation. The external validation cohort was derived from splitting different samples and multicenter studies. The detailed information above is shown in **Table 3**.

**Table 3:** **S**ummary of the publications included in the systematic review, highlighting paper information and nomogram prediction outcome (LLNM).

Author	Year	Period		Type	Source data	Outcome	Total sample enrolled	Statistical model	Validation method	Sample size (training)	Sample size (InV	Sample size (ExV
Chang, Q	2022 (33)	2018.8-2021.11		D/V	RC	LLNM	1,324	Logistic regression	Split-sample V (3:1) +EXV	993	331	631
Dong, L	2023	2015.9-2020.12		D/V	MC	LLNM	1,213	Logistic regression	Split-sample V (7:3) +EXV	800	345	68
Dou, Y	2020 (40)	2016.1-2017.12		D/V	MC	LLNM-II	653	Logistic regression	NI	460	193	NI
Dou, Y	2020 (40)	2016.1-2017.12		D/V	MC	LLNM-III + IV	653	Logistic regression	NI	460	193	NI
Feng, J	2022	2019.3-2020.5		D	RC	LLNM	528	Logistic regression	Bootstrap resampling	528	Bootstrap resampling	NI
Feng, J	2022	2019.3-2020.5		D	RC	mLLNM	528	Logistic regression	Bootstrap resampling	528	Bootstrap resampling	NI
Feng, J	2022	2019.1-2022.1		D	RC	LLNM	1106	Logistic regression	Bootstrap resampling	1,106	Bootstrap resampling	NI
Heng, Y	2020 (42)	2017-2019		D/V	PC + MC	LLNM	434	Logistic regression	Bootstrap resampling	434	1,000 bootstrap resamples	NI
Liu, S	2021 (43)	2016.1-2018.12		D	RC	LLNM	1,198	Logistic regression	NI	1,198	NI	NI
Liu, W	2023 (35)	2011.2-2022.4		D/V	MC	LLNM	6,650	Logistic regression	Split-sample V (7:3)	4,247	1,821	582
Ma, Y	2023 (44)	2019.1-2021.12		D/V	RC	LLNM	336	Logistic regression	Split-sample V (7:3)	228	108	NI
Tong, Y	2021 (41)	2018.2-2018.12		D/V	RC	LLNM	868	Logistic regression	NI	600	286	NI
Tong, Y	2022 (32)	2019.1-2019.6		D/V	MC	LLNM	720	Logistic regression	MC	300	143	133
Wang, J	2023 (45)	2015-2018		D/V	RC	LLNM	476	LASSO + logistic regression	Split-sample V (3:1)	355	121	NI
Zhao, L	2022 (46)	2013.1-2021.6		D	RC	LLNM	873	Logistic regression	Split-sample by admission	702	NI	171
Zhu, J	2023	2013.1-2018.6		D/V	MC	LLNM	2,612	Logistic regression	Split-sample V (3:1) +EXV	1,732	578	302
Zhuo, X	2022 (47)	2013.1-2019.6		D/V	RC	LLNM	253	Logistic regression	Split-sample by admission	138	115	NI
Zou, Y	2021 (48)	2015.7-2019.6		D/V	RC	LLNM	507	Logistic regression	Split-sample V (7:3)	280	126	101
Gong, J	2023 (49)	2011-2021		D	RC	LLNM	2166	Logistic regression	NI	2,166	NI	NI
Huang, C	2022	2016.1-2018.12		D	RC	LLNM-II	418	Logistic regression	NI	418	NI	NI

D, develop model; V, validate model; RC, retrospective cohort study; MC, multicenter cohort study; mLLNM, multiple lateral lymph node metastasis; NI, no information.

### 3.2 Risk of bias assessment in included studies

**Figure 2:**
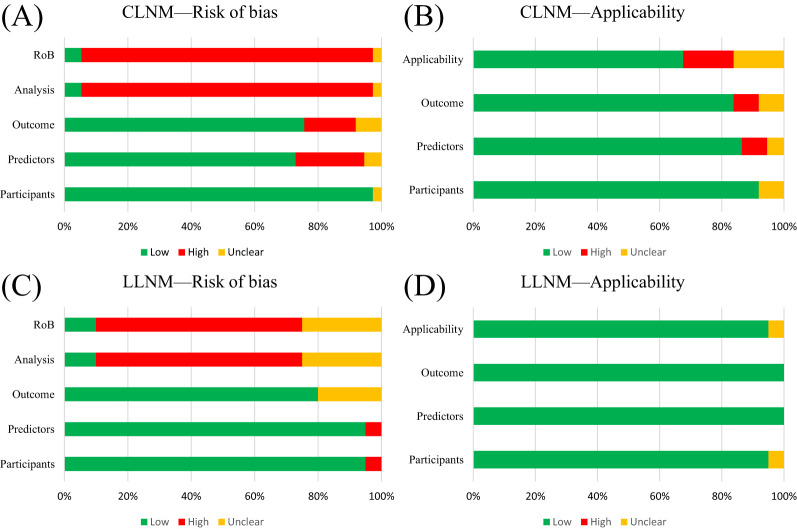
PROBAST summary for all nomogram development. **(A)** PROBAST risk of bias for CLNM included models. **(B)** Applicability of those included in the CLNM model systematic review. **(C)** PROBAST risk of bias for LLNM included models. **(D)** Applicability of those included in LLNM model systematic review.

For CLNM group training studies (**Table 4**, **Figures 2A, B**), the overall risk of bias (ROB) was high in 34 out of 37 studies, primarily due to the unreliability of the analysis domain (34/37). Additionally, the data sources and inclusion/exclusion criteria of the studies also posed a high risk of bias. Applicability addresses how relevant the included articles are to the review questions. The overall applicability of development studies was unclear in six out of 37 studies, mainly because of inconsistencies in participant domains (3/37), predictors (2/37), and outcomes (3/37).

**Table 4:** PROBAST risk of bias of the CLNM group.

Information	Year	Outcome	Development study								
			Risk of bias (-/?/+)				Applicability (-/?/+)			Overall (-/?/+)	
			Participants	Predictors	Outcome	Analysis	Participants	Predictors	Outcome	ROB	Applicability
Chang, L	2023	CLNM	+	+	+	–	+	+	+	–	+
Chang, Q	2022 (33)	CLCM	+	+	+	+	+	+	+	+	+
Chen, F	2023	CLNM	+	+	+	–	?	+	+	–	?
Chen, H	2024	CLNM	+	+	+	–	+	+	+	–	+
Chen, Q	2023	CLNM	+	–	+	–	+	–	+	–	–
Dai, Q	2022 (34)	CLNM	+	–	–	–	+	+	?	–	?
Dai, Q	2022 (34)	CLNM	+	–	–	–	?	+	?	–	?
Deng, Y	2023	CLNM	+	?	+	–	+	+	+	–	+
Du, J	2023	CLNM	+	+	+	–	+	+	+	–	+
Feng, J W	2021	CLNM	+	+	+	–	+	+	+	–	+
Feng, J W	2024 (50)	CLNM	+	?	?	–	+	?	+	–	?
Gao, X	2021	CLNM	+	–	+	–	+	–	+	–	–
He, L	2024	CLNM	+	–	+	–	+	+	+	–	+
Hei, H	2023	CLNM	+	+	+	–	?	+	–	–	–
Hu, Q	2021	CLNM	+	+	+	–	+	+	+	–	+
Hu, W	2023	CLNM	+	–	+	–	+	+	+	–	+
Huang, C	2020	CLNM	+	+	+	–	+	+	+	–	+
Huang, Y	2023	CLNM	+	–	+	–	+	–	+	–	–
Jiang, L	2023	CLNM	+	+	+	–	+	+	+	–	+
Li, J	2022	CLNM	?	+	+	–	+	+	+	–	+
Lin, P	2021	CLNM	+	+	?	–	+	+	?	–	?
Liu, W	2023 (35)	CLNM	+	+	+	?	+	+	+	?	+
Pang, J	2023 (36)	CLNM	+	+	+	–	+	+	+	–	+
Qiao, D	2024	CLNM	+	+	+	–	+	+	+	–	+
Song, X	2024	CLNM	+	+	+	–	+	+	+	–	+
Sun, F	2021 (37)	CLNM	+	+	+	+	+	+	+	+	+
Wang, Z	2022	CLNM	+	+	+	–	+	+	+	–	+
Wei, L	2024	CLNM	+	+	+	–	+	+	+	–	+
Wen, Q	2022	CLNM	+	–	+	–	+	+	+	–	+
Xue, J	2023	CLNM	+	+	–	–	+	+	–	–	–
Xue, T	2021	CLNM	+	+	–	–	+	+	–	–	–
Yang, Z	2020 (38)	CLNM	+	+	–	–	+	?	+	–	?
Zeng, B	2022 (39)	CLNM	+	+	+	–	+	+	+	–	+
Zhang, H	2020	CLNM	+	+	–	–	+	+	+	–	+
Zhou, S C	2020	CLNM	+	+	+	–	+	+	+	–	+
Tong, Y	2022 (32)	CLNM	+	+	?	–	+	+	+	–	+
Zhao, D	2024	CLNM	+	+	+	–	+	+	+	–	+

+, low risk; ?, unclear; -, high risk.

For the LLNM group development studies (**Table 5**, **Figures 2C, D**), the overall risk of bias (ROB) was high in 13 out of 20 due to incompatibilities in those analysis domains. The overall applicability of development studies was low risk in 19 out of 20. The articles included in the LLNM group addressed the review questions. Overall, the applicability of the LLNM group development studies was good.

**Table 5:** PROBAST risk of bias of the LLNM group.

Information	Year	Outcome	Development study								
			Risk of bias (-/?/+)				Applicability (-/?/+)			Overall (-/?/+)	
			Participants	Predictors	Outcome	Analysis	Participants	Predictors	Outcome	ROB	Applicability
Chang, Q	2022 (33)	LLCM	+	+	+	?	+	+	+	?	+
Dong, L	2023	LLCM	+	+	+	–	+	+	+	–	+
Dou, Y	2020 (40)	LLNM-II	+	+	+	–	+	+	+	–	+
Dou, Y	2020 (40)	LLNM-III + IV	+	+	+	–	+	+	+	–	+
Feng, J W	2022	LLNM	+	+	+	–	+	+	+	–	+
Feng, J W	2022	mLLNM	+	+	+	–	+	+	+	–	+
Feng, J W	2022	LLNM	+	+	?	–	+	+	+	–	+
Heng, Y	2020 (42)	LLNM	+	+	+	?	+	+	+	?	+
Liu, S	2021 (43)	LLNM	+	+	+	–	+	+	+	–	+
Liu, W	2023 (35)	LLNM	+	+	+	?	+	+	+	?	+
Ma, Y	2023 (44)	LLNM	+	+	+	+	+	+	+	+	+
Tong, Y	2021 (41)	LLNM	+	+	+	–	+	+	+	–	+
Tong, Y	2022 (32)	LLNM	+	+	?	–	+	+	+	–	+
Wang, J	2023 (45)	LLNM	+	+	?	–	+	+	+	–	+
Zhao, L	2022 (46)	LLNM	+	+	+	?	+	+	+	?	+
Zhu, J	2023	LLNM	+	+	+	+	+	+	+	+	+
Zhuo, X	2022 (47)	LLNM	+	+	+	–	+	+	+	–	+
Zou, Y	2021 (48)	LLNM	+	+	+	?	+	+	+	?	+
Gong, J	2023 (49)	LLNM	–	–	?	–	?	+	+	–	?
Huang, C	2022	LLNM-II	+	+	+	–	+	+	+	–	+

+, low risk; ?, unclear; -, high risk.

The predictive model, which lacked discussion on model fitting, calibration evaluation, and DCA curves, resulted in a high risk of bias (ROB) for the analysis domain. Furthermore, most validation studies did not report missing data or update the nomograms. Meanwhile, the assessment of risk stratification was carried out on a blank board. We also conducted PROBAST ROB and TRIPOD + AI assessments of external validation cohort studies to visually present the results. The detailed table is available in **Supplementary Material S4**.

### 3.3 Nomogram predictors

#### 3.3.1 CLNM group

**Figure 3:**
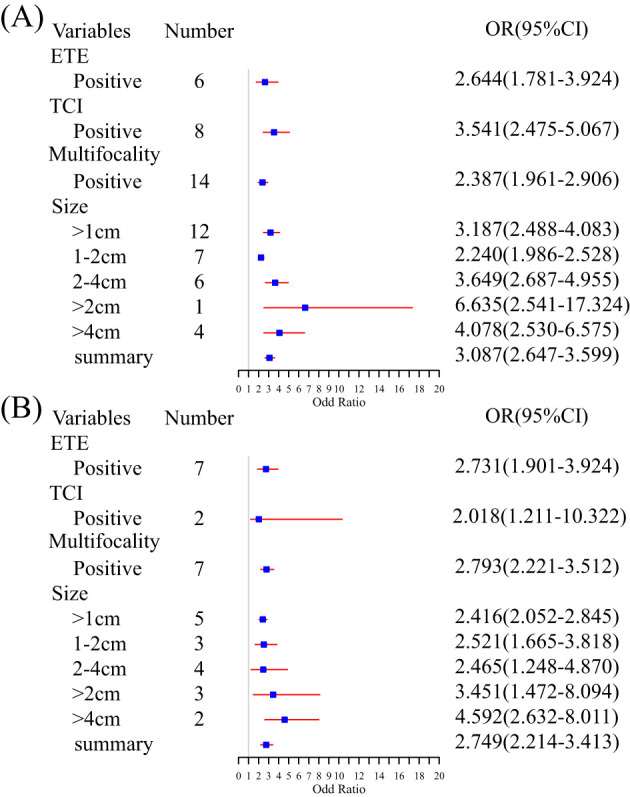
Summary meta-analysis forest plot for several standard predictor variables: **(A)** subgroup analysis of risk factors for CLNM and **(B)** subgroup analysis of risk factors for LLNM.

In most studies, age, gender, tumor diameters or size, location, multifocality, and capsular invasion or ETE were analyzed. We examined risk factors by collecting standard categorical variables from the included literature. ETE (OR, 95% CI, 2.644, 2.644, 1.781–3.924), TCI (3.541, 3.541, 2.475–5.067), multifocality (2.387, 2.387, 1.961–2.90), and tumor size (3.087, 3.087, 2.647–3.599) are identified risk factors for CLNM (**Figure 3A**). All nomograms were constructed using multivariate logistic regression. Overall, four and 22 factors were included in the univariate and multivariate analyses, respectively. A minimum of three (32) predictors and a maximum of 10 (36) predictors were displayed in the nomograms. We summarized the frequency of common risk factors (**Supplementary Material S5**). Interestingly, almost all studies incorporated US information. Some studies adopted LASSO regression based on image features to provide more details compared to the malignant features observed in ultrasound of TC. Some studies obtained combined scores by integrating relevant characteristics and risk factors. The laboratory tests used in the nomograms mainly consisted of thyroid function tests. For complete details of the risk factors for the CLNM-group nomograms, see **Supplementary Materials S5** and **S6**.

#### 3.3.2 LLNM group

Age, gender, tumor size or diameter, central lymph node status, location, and multifocality were analyzed in all included studies. ETE (OR, 95% CI, 2.731, 2.731, 1.901–3.924), TCI (2.018, 2.018, 1.211–10.322), and multifocality (2.793, 2.793, 2.221–3.512) were examined. An increasing tumor diameter suggested a high risk of LNM (>1 cm vs. >2 cm vs. >4 cm, 2.416, 2.416, 2.052–2.845 vs. 3.451, 3.451, 1.472–8.094 vs. 4.592, 4.592, 2.632–8.011) (**Figure 3B**). Multivariate logistic regression analysis was used in all models to identify independent risk factors. A minimum of two (46) and a maximum of 11 predictors (45) were shown in nomograms. The image features from the US and CT allowed many predictors to be displayed in the nomograms. Although laboratory indicators were included in five studies, only four predictors, such as PS-Tg ≥30.175 ng/mL, serum TSH ≥ 2.910 uIU/mL, SIC > 45 Ug/L, and SII (33, 35, 45, 46) were verified as independent risk factors. For complete details of the risk factors for LLNM group nomograms, see **Supplementary Materials S5** and **S6**.

#### 3.3.3 Nomogram performance

The C-index for developing CLNM studies ranged from 0.703 (39) to 0.960 (95% CI, 0.947–0.972) (50), with the majority concentrated in the 0.75–0.9 range, suggesting good model prediction performance (C-index >0.7). The C-index of the external validation studies ranged from 0.734 (35) to 0.923 (95% CI, 0.893–0.947) (37). Although 28 studies reported the DCA analysis, its efficacy was challenging to accept. Almost all studies provided calibration curves. However, few studies reported calibration performance and *p*-values. All studies with external validation included calibration curves as well as DCA, yet few studies reported calibrated slopes and/or intercepts, making it difficult to gather data for analysis.

The C-index of the developed LLNM nomograms ranged from 0.702 (95% CI, 0.667–0.736) (49) to 0.956 (95% CI, 0.925–0.986) (47). The internal validation C-index ranged from 0.731 (95% CI, 0.635–0.827) (44) to 0.914 (95% CI: 0.842–0.987) (41). The C-index of the external validation studies ranged from 0.762 (35) to 0.915 (95% CI, 0.862–0.967) (47). The prediction performance of the developed nomogram was good (>0.7). Calibration was performed in 18 studies, and 12 studies reported DCA. Compared to the external validation of the nomogram for the CLNM group, all LLNM groups with external validation provided calibration plots (regardless of internal or external validation), and only one did not assess the DCA curves. Few of the included studies reported calibrated slopes and/or intercepts, while none reported observed/expected ratios, making it challenging to assess in a meta-analysis and validate quantitatively in the clinical cohort. **Supplementary Material S7** summarizes the performance and calibration of the nomograms.

### 3.4 Meta-analysis

**Figure 4:**
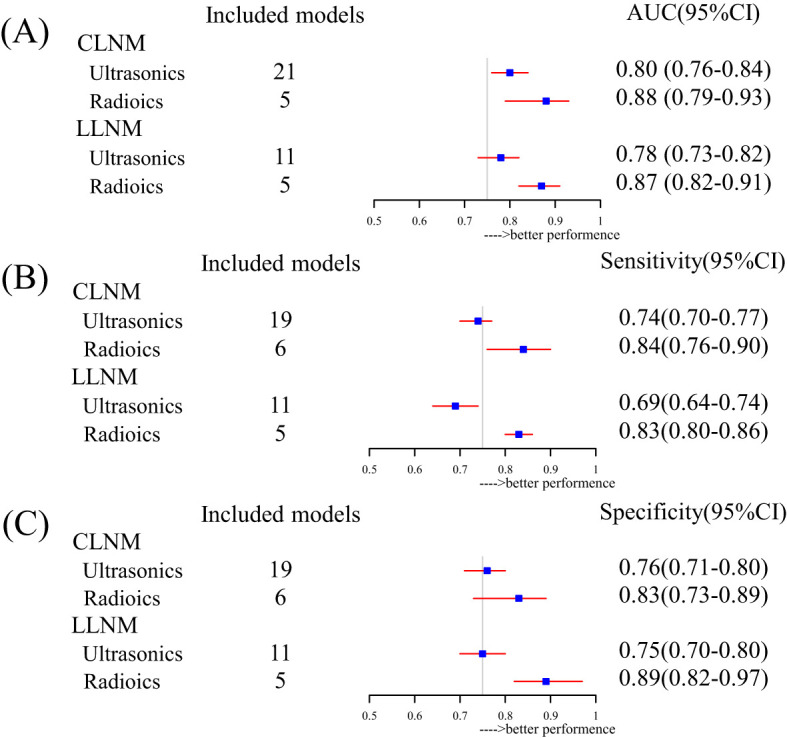
Subgroup summary evaluation of model efficacy for different outcomes: **(A)** forest plot for pooled AUC based on inclusion factor classification, **(B)** forest plot for pooled sensitivity based on inclusion factor classification, and **(C)** forest plot for pooled specificity based on inclusion factor classification.

We performed a subgroup analysis based on model characteristics. Models that included biochemical tests and non-uniform metrics demonstrated greater heterogeneity, which led to their exclusion. Ultrasound was the most frequently incorporated feature in the models. The pooled C-index for CLNM-US and LLNM-US was 0.80 (95% CI, 0.76–0.84) and 0.78 (0.73–0.82), respectively (**Figure 4A**). The ultrasound model for predicting CLNM showed excellent performance, while the 95% CI for LLNM-US was 0.73 (less than 0.75), indicating moderate predictive efficacy. The model combined with radiomics enhanced the effectiveness of the CLNM-RS and LLNM-RS summary C-index, which were 0.88 (0.79–0.93) and 0.90 (0.83–0.95) (**Figure 4A**), respectively, both demonstrating excellent predictive efficacy. We collected and carried out a subgroup analysis for sensitivity and specificity in the included models. Similarly, both outcomes combined with the radiomics group exhibited better performance in sensitivity and specificity (**Figures 4B, C**). A comprehensive evaluation of multi-source detection methods proved more conducive to detecting LNM. Additionally, the external validation group models demonstrated excellent predictive efficacy (**Supplementary Material S8**). However, in the CLNM EX group, the combination of four nomograms resulted in wide 95% prediction intervals, indicating the impact of random error and uncertainty in the model parameters. All of the detailed information can be found in **Supplementary Materials S7** and **S8**.

## 4 Discussion

The nomogram model serves as a visual tool for surgeons in their decision-making process. However, as of the submission of this article, no official guidelines exist for grading and applying predictive models. In our work, we performed a systematic review and meta-analysis of nomograms used to predict LNM in PTC within a real-world Chinese context. We systematically summarized the nomograms based on clinical data, identified those suitable for further research, provided personalized recommendations for individual patients, and offered valuable reference tools for clinical practice.

Based on the inclusion and exclusion criteria, 57 nomograms of predictive models were selected. The results of this review demonstrate that most predictive models were single-center retrospective studies and were modeled and validated simultaneously. Recently, a meta-study has shown that the model performance based on logistic regression was considered satisfactory (51). Univariate and multivariate logistic regression analyses are the most common methods for screening variables and constructing models in this review. The CLNM rate of PTC is more than 50% (52, 53), and the LLNM rate is approximately 20% (54). The included research on the LNM rate was broadly consistent with the conclusions.

We summarized the frequency of standard variables included in the review. Age and tumor size or diameter were the most common independent risk factors. Younger patients are at a high risk for LNM, and the findings confirmed the previous conclusions (34, 42). Nevertheless, there is controversy over the age demarcation between 45 and 55 years. The difference in clinical staging between the seventh and eighth editions of the AJCC guidelines has led to this debate. Few studies have focused on the population within this interval (7, 8). Previous research demonstrated a positive correlation between tumor diameters and the risk of LNM; tumor size indicated tumor aggressiveness (55). However, the AJCC guidelines emphasize age and distant metastasis-oriented TNM staging criteria (7). Multifocality, capsular invasion, and ETE have been verified by many studies to be positively correlated with thyroid LNM (38, 43, 48), consistent with the results of our pooled meta-analysis (**Figure 3**). Simultaneously, some studies have suggested a potential correlation with prognosis (56, 57). Some predictive models incorporated biochemical analysis, such as Tg, Tg-Ab, and SII (33, 35, 45, 46), but they were limited in their ability to predict metastasis and were primarily used to study prognosis. High-risk pathological subtypes, lymph node metastasis count and size, and extranodal extension have been regarded as risk factors associated with disease recurrence and prognosis as supported by studies (58–60). Nonetheless, due to the limitations of the original research, most data failed to adequately cover these key variables. Novel and effective biomarkers are needed for PTC and LNM diagnosis.

In contrast to Maria’s study (61), random-effects models were used for this meta-analysis to explain heterogeneity. The meta-analysis findings validate that ultrasound has moderate to good predictive efficacy (pooled AUC, sensitivity, and specificity) for CLNM, which is better than LLNM (**Figure 4**). The model’s efficacy and the incorporation of radiomics were greater than that of the single model (**Figure 4**). Multi-omics predictive models have better predictive performance than a single model. Similarly, Liu’s study identified logistic regression as the best method for LNM of TC models (51). More importantly, our analysis highlights the overall potential of nomogram models as predictive tools for LNM in TC and the need for further research efforts. However, in many predictive models, the absence of external validation is a prevalent issue that greatly hinders the generalization of their findings and deters the application of nomograms in clinical practice.

The present study shows that 34/37 and 13/20 of the overall ROB of included studies had a high risk, respectively (**Figure 2**, **Tables 4**, **5**). We reviewed the reasons and limitations of the high-risk studies. First, the participants’ domain, comprising the majority of populations included in this systematic review, derived from single-central or multicentral retrospective cohort studies, which made it challenging to avoid selection bias. Additionally, predictors predominantly came from clinical detection or composite indicators. Converting continuous variables to categorical variables according to the guidelines, such as age or tumor size, neglected the distribution characteristics of these variables, which, in turn, increased the risk of information bias. In the outcome domain, the results from the included studies were not entirely uniform, and differences in stage and LNM region could affect the performance of pooled models. Most studies reported calibration curves, whereas only a minority included Hosmer–Lemeshow test results and calibration metrics, which may lead to an overestimation of accurate event rates. We were unable to assess the overall calibration of the nomograms, and evaluating the degree of model fit was challenging in the literature.

The significant diversity among nomograms, combined with a serious shortage of external validation studies, has limited its clinical application (62, 63). Consequently, it remains uncertain whether and how patients and clinicians could benefit from using nomograms. Ultimately, although the initial search strategy identified 2,835 articles, all non-Chinese studies did not meet the inclusion criteria, possibly due to the relatively low proportion of relevant studies in other countries. Nonetheless, we present the heat map of China, intending to recommend that local doctors adopt models appropriate for the level of diagnosis and treatment. This work provides new insights and perspectives. With the rapid advancement of various model algorithms and artificial intelligence applications in hospitals, nomograms, as an early approach for model integration, have gained widespread acceptance. Furthermore, the development of web-based prediction models enhances their broader application in clinical practice. Influenced by artificial intelligence, integrating models with electronic medical records (EMRs) not only improves usability but also significantly boosts the accuracy of predictions. Integrating models into electronic health records or clinical pathways would be beneficial.

Multi-centric data and comprehensive validation methods are crucial to develop predictive models. A significant number of validations of the same model enhance its effectiveness for future quantitative analysis. This study aims to identify gaps in predictive model research, particularly emphasizing the lack of established guidelines. Given the potential for bias in many studies, clinical decision-making would benefit from the incorporation of prospective validation studies to support more robust and reliable conclusions. In this context, prospective cohort studies were conducted to improve the level of evidence and provide visual reference tools and new insights for clinicians and medical guidelines in China.

## 5 Conclusions

The feature-combined model is better suited for use in Chinese clinical practice than the LNM of PTC. Despite demonstrating excellent discrimination accuracy of prediction models, significant heterogeneity has limited the application of uninformed predictive nomograms. At the same time, the systematic review offers reference tools for physicians to choose appropriate nomograms based on individual clinical needs in China. Future research should focus on external validation and assessment of clinical utility to effectively translate the nomogram into clinical practice rather than solely for research purposes.
