# Supplementary material for: Precision of predictive nomograms for lymph node metastasis of thyroid cancer from Chinese real-world study: a systematic review and meta-analysis
Source: Front Endocrinol (Lausanne). 2025 Jul 9;16:1617563. doi: 10.3389/fendo.2025.1617563 (PMC12283272; doi:10.3389/fendo.2025.1617563)
Supplement: Supplementary file 1 [file DataSheet1.docx]

**Precision of predictive nomograms for lymph node metastasis of thyroid cancer from** **Chinese real-world study: a systematic review and meta-analysis**

Yongke Wu^†^, Yuanhao Su^†^, Yiyuan Zhao, Nassuf Mourdi, Zhidong Wang*

Department of Geriatric General Surgery, The Second Affiliated Hospital, Xi'an Jiaotong University, Xi'an 710004, Shaanxi, China

*Corresponding author:

Zhidong Wang, Department of Geriatric General Surgery, The Second Affiliated Hospital, Xi'an Jiaotong University, 157 West 5th Road, Xi'an 710004, Shaanxi, China. Email: [xawzd@163.com](mailto:xawzd@163.com)(Z.D.W.)

Supplemental Information Comtent

[Supplemental material 1—Search formula 3](#_Toc186407106)

[Pubmed: Medical Subject Headings& Entry terms 3](#_Toc186407107)

[Scopus: 5](#_Toc186407108)

[Web of science: 5](#_Toc186407109)

[Supplemental material 2—included studies in this systematic review 7](#_Toc186407110)

[CLNM group 7](#_Toc186407111)

[LLNM group 9](#_Toc186407112)

[Supplemental material 3—China heatmap of predictive model of PTC-LNM 11](#_Toc186407113)

[Supplemental material 4—PROBAST risk of bias for external validation 14](#_Toc186407114)

[Supplemental material 5—Frequency of risk factors 16](#_Toc186407115)

[Supplemental material 6—Risk factors of nomogram 17](#_Toc186407116)

[Table 1 Risk variables of CLNM group nomograms 17](#_Toc186407117)

[Table 2 Risk variables of LLNM group nomograms 23](#_Toc186407118)

[Supplemental material 7—AUC of included model 26](#_Toc186407119)

[Table 1 Summarize the performance and calibration of the CLNM-group nomograms 26](#_Toc186407120)

[Table 2 Summarize the performance and calibration of the LLNM-group nomograms 32](#_Toc186407121)

[Supplemental material 8—Pooled summary model power 36](#_Toc186407122)

[Figure 1 Subgroup meta-analysis of model pooled C-index 36](#_Toc186407123)

[Table 1 Subgroup meta-analysis of model pooled C-index 37](#_Toc186407124)

[Table 2 Subgroup meta-analysis of model pooled sensitivity 37](#_Toc186407125)

[Table 3 Subgroup meta-analysis of model pooled specificity 37](#_Toc186407126)

# Supplemental material 1—Search formula

A systematic search strategy was performed in PubMed, Web of Science, and Scopus without language restrictions using the following keywords: (thyroid cancer OR differentiated thyroid cancer OR papillary thyroid carcinoma) AND (nomogram OR nomograms) AND (CLNM OR LLNM OR central lymph node metastasis OR lateral lymph node metastasis). In order to search the literature in a more detailed way, we adopted the search method of combining the medical subject headings with the entry and terms.

## Pubmed: Medical Subject Headings& Entry terms

("Thyroid Neoplasms"[MeSH Terms] OR ("Thyroid Neoplasms"[MeSH Terms] OR ("thyroid"[All Fields] AND "neoplasms"[All Fields]) OR "Thyroid Neoplasms"[All Fields] OR ("Thyroid Neoplasms"[MeSH Terms] OR ("thyroid"[All Fields] AND "neoplasms"[All Fields]) OR "Thyroid Neoplasms"[All Fields] OR ("neoplasm"[All Fields] AND "thyroid"[All Fields]) OR "neoplasm thyroid"[All Fields]) OR ("Thyroid Neoplasms"[MeSH Terms] OR ("thyroid"[All Fields] AND "neoplasms"[All Fields]) OR "Thyroid Neoplasms"[All Fields] OR ("thyroid"[All Fields] AND "neoplasm"[All Fields]) OR "thyroid neoplasm"[All Fields]) OR ("Thyroid Neoplasms"[MeSH Terms] OR ("thyroid"[All Fields] AND "neoplasms"[All Fields]) OR "Thyroid Neoplasms"[All Fields] OR ("neoplasms"[All Fields] AND "thyroid"[All Fields]) OR "neoplasms thyroid"[All Fields]) OR ("Thyroid Neoplasms"[MeSH Terms] OR ("thyroid"[All Fields] AND "neoplasms"[All Fields]) OR "Thyroid Neoplasms"[All Fields] OR ("thyroid"[All Fields] AND "carcinoma"[All Fields]) OR "thyroid carcinoma"[All Fields]) OR ("Thyroid Neoplasms"[MeSH Terms] OR ("thyroid"[All Fields] AND "neoplasms"[All Fields]) OR "Thyroid Neoplasms"[All Fields] OR ("carcinomas"[All Fields] AND "thyroid"[All Fields]) OR "carcinomas thyroid"[All Fields]) OR ("Thyroid Neoplasms"[MeSH Terms] OR ("thyroid"[All Fields] AND "neoplasms"[All Fields]) OR "Thyroid Neoplasms"[All Fields] OR ("carcinoma"[All Fields] AND "thyroid"[All Fields]) OR "carcinoma thyroid"[All Fields]) OR ("Thyroid Neoplasms"[MeSH Terms] OR ("thyroid"[All Fields] AND "neoplasms"[All Fields]) OR "Thyroid Neoplasms"[All Fields] OR ("thyroid"[All Fields] AND "carcinomas"[All Fields]) OR "thyroid carcinomas"[All Fields]) OR ("Thyroid Neoplasms"[MeSH Terms] OR ("thyroid"[All Fields] AND "neoplasms"[All Fields]) OR "Thyroid Neoplasms"[All Fields] OR ("cancer"[All Fields] AND "thyroid"[All Fields]) OR "cancer of the thyroid"[All Fields]) OR ("Thyroid Neoplasms"[MeSH Terms] OR ("thyroid"[All Fields] AND "neoplasms"[All Fields]) OR "Thyroid Neoplasms"[All Fields] OR ("cancer"[All Fields] AND "thyroid"[All Fields]) OR "cancer of thyroid"[All Fields]) OR ("Thyroid Neoplasms"[MeSH Terms] OR ("thyroid"[All Fields] AND "neoplasms"[All Fields]) OR "Thyroid Neoplasms"[All Fields] OR ("thyroid"[All Fields] AND "cancers"[All Fields]) OR "thyroid cancers"[All Fields]) OR ("Thyroid Neoplasms"[MeSH Terms] OR ("thyroid"[All Fields] AND "neoplasms"[All Fields]) OR "Thyroid Neoplasms"[All Fields] OR ("thyroid"[All Fields] AND "cancer"[All Fields]) OR "thyroid cancer"[All Fields]) OR ("Thyroid Neoplasms"[MeSH Terms] OR ("thyroid"[All Fields] AND "neoplasms"[All Fields]) OR "Thyroid Neoplasms"[All Fields] OR ("cancers"[All Fields] AND "thyroid"[All Fields]) OR "cancers thyroid"[All Fields]) OR ("Thyroid Neoplasms"[MeSH Terms] OR ("thyroid"[All Fields] AND "neoplasms"[All Fields]) OR "Thyroid Neoplasms"[All Fields] OR ("cancer"[All Fields] AND "thyroid"[All Fields]) OR "cancer thyroid"[All Fields]) OR ("Thyroid Neoplasms"[MeSH Terms] OR ("thyroid"[All Fields] AND "neoplasms"[All Fields]) OR "Thyroid Neoplasms"[All Fields] OR ("thyroid"[All Fields] AND "adenoma"[All Fields]) OR "thyroid adenoma"[All Fields]) OR ("Thyroid Neoplasms"[MeSH Terms] OR ("thyroid"[All Fields] AND "neoplasms"[All Fields]) OR "Thyroid Neoplasms"[All Fields] OR ("adenomas"[All Fields] AND "thyroid"[All Fields]) OR "adenomas thyroid"[All Fields]) OR ("Thyroid Neoplasms"[MeSH Terms] OR ("thyroid"[All Fields] AND "neoplasms"[All Fields]) OR "Thyroid Neoplasms"[All Fields] OR ("adenoma"[All Fields] AND "thyroid"[All Fields]) OR "adenoma thyroid"[All Fields]) OR ("Thyroid Neoplasms"[MeSH Terms] OR ("thyroid"[All Fields] AND "neoplasms"[All Fields]) OR "Thyroid Neoplasms"[All Fields] OR ("thyroid"[All Fields] AND "adenomas"[All Fields]) OR "thyroid adenomas"[All Fields]))) AND ("lymphatic metastasis"[MeSH Terms] OR ("lymphatic"[All Fields] AND "metastasis"[All Fields]) OR "lymphatic metastasis"[All Fields] OR ("lymphatic metastasis"[MeSH Terms] OR ("lymphatic"[All Fields] AND "metastasis"[All Fields]) OR "lymphatic metastasis"[All Fields] OR ("lymphatic"[All Fields] AND "metastases"[All Fields]) OR "lymphatic metastases"[All Fields]) OR ("lymphatic metastasis"[MeSH Terms] OR ("lymphatic"[All Fields] AND "metastasis"[All Fields]) OR "lymphatic metastasis"[All Fields] OR ("lymph"[All Fields] AND "node"[All Fields] AND "metastasis"[All Fields]) OR "lymph node metastasis"[All Fields]) OR ("lymphatic metastasis"[MeSH Terms] OR ("lymphatic"[All Fields] AND "metastasis"[All Fields]) OR "lymphatic metastasis"[All Fields] OR ("lymph"[All Fields] AND "node"[All Fields] AND "metastases"[All Fields]) OR "lymph node metastases"[All Fields]) OR ("lymphatic metastasis"[MeSH Terms] OR ("lymphatic"[All Fields] AND "metastasis"[All Fields]) OR "lymphatic metastasis"[All Fields] OR ("metastasis"[All Fields] AND "lymph"[All Fields] AND "node"[All Fields]) OR "metastasis lymph node"[All Fields]) OR (("central"[All Fields] OR "centrally"[All Fields] OR "centrals"[All Fields]) AND ("lymphatic metastasis"[MeSH Terms] OR ("lymphatic"[All Fields] AND "metastasis"[All Fields]) OR "lymphatic metastasis"[All Fields] OR ("lymph"[All Fields] AND "node"[All Fields] AND "metastasis"[All Fields]) OR "lymph node metastasis"[All Fields])) OR (("functional laterality"[MeSH Terms] OR ("functional"[All Fields] AND "laterality"[All Fields]) OR "functional laterality"[All Fields] OR "laterality"[All Fields] OR "lateral"[All Fields] OR "lateralisation"[All Fields] OR "lateralisations"[All Fields] OR "lateralise"[All Fields] OR "lateralised"[All Fields] OR "lateralises"[All Fields] OR "lateralising"[All Fields] OR "lateralities"[All Fields] OR "lateralization"[All Fields] OR "lateralizations"[All Fields] OR "lateralize"[All Fields] OR "lateralized"[All Fields] OR "lateralizes"[All Fields] OR "lateralizing"[All Fields] OR "laterally"[All Fields] OR "laterals"[All Fields]) AND ("lymphatic metastasis"[MeSH Terms] OR ("lymphatic"[All Fields] AND "metastasis"[All Fields]) OR "lymphatic metastasis"[All Fields] OR ("lymph"[All Fields] AND "node"[All Fields] AND "metastasis"[All Fields]) OR "lymph node metastasis"[All Fields])) OR "CLNM"[All Fields] OR "LLNM"[All Fields]) AND ("nomogram s"[All Fields] OR "nomograms"[MeSH Terms] OR "nomograms"[All Fields] OR "nomogram"[All Fields] OR ("nomogram s"[All Fields] OR "nomograms"[MeSH Terms] OR "nomograms"[All Fields] OR "nomogram"[All Fields]))

## Scopus:

( ( ALL ( lymphatic AND metastasis ) OR ALL ( lymphatic AND metastases ) OR ALL ( lymph AND node AND metastasis ) OR ALL ( lymph AND node AND metastases ) OR ALL ( metastasis, AND lymph AND node ) OR ALL ( central AND lymph AND node AND metastasis ) OR ALL ( clnm ) OR ALL ( lateral AND lymph AND node AND metastasis ) OR ALL ( llnm ) ) ) AND ( ( ALL ( nomogram ) AND ALL ( nomograms ) ) ) AND ( ( ALL ( thyroid AND neoplasms ) OR ALL ( neoplasm, AND thyroid ) OR ALL ( thyroid AND neoplasm ) OR ALL ( neoplasms, AND thyroid ) OR ALL ( thyroid AND carcinoma ) OR ALL ( carcinomas, AND thyroid ) OR ALL ( carcinoma, AND thyroid ) OR ALL ( thyroid AND carcinomas ) OR ALL ( cancer AND of AND the AND thyroid ) OR ALL ( cancer AND of AND thyroid ) OR ALL ( thyroid AND cancers ) OR ALL ( thyroid AND cancer ) OR ALL ( cancers, AND thyroid ) OR ALL ( cancer, AND thyroid ) OR ALL ( thyroid AND adenoma ) OR ALL ( adenomas, AND thyroid ) OR ALL ( adenoma, AND thyroid ) OR ALL ( thyroid AND adenomas ) ) )

## Web of science:

<https://webofscience.clarivate.cn/wos/alldb/summary/e1302abd-786d-47f8-b4be-f0446b78ac8d-fadc4702/relevance/1>

Query #1

**Thyroid Neoplasms** (Topic) or **Neoplasm, Thyroid** (Topic) or **Thyroid Neoplasm** (Topic) or **Neoplasms, Thyroid** (Topic) or **Thyroid Carcinoma** (Topic) or **Carcinomas, Thyroid** (Topic) or **Carcinoma, Thyroid** (Topic) or **Thyroid Carcinomas** (Topic) or **Cancer of the Thyroid** (Topic) or **Cancer of Thyroid** (Topic) or **Thyroid Cancers** (Topic) or **Thyroid Cancer** (Topic) or **Cancers, Thyroid** (Topic) or **Cancer, Thyroid** (Topic) or **Thyroid Adenoma** (Topic) or **Adenomas, Thyroid** (Topic) or **Adenoma, Thyroid** (Topic) or **Thyroid Adenomas** (Topic) and **Preprint Citation Index** (Exclude – Database)

Edit

Query #2

**Lymphatic Metastasis** (Topic) or **Lymphatic Metastases** (Topic) or **Lymphatic Metastases** (Topic) or **Lymph Node Metastases** (Topic) or **Metastasis, Lymph Node** (Topic) or **central lymph node metastasis** (Topic) or **CLNM** (Topic) or **lateral lymph node metastasis** (Topic) or **LLNM** (Topic) and **Preprint Citation Index** (Exclude – Database)

Edit

Query #3

**nomogram** (Topic) or **nomograms** (Topic) and **Preprint Citation Index** (Exclude – Database)

# Supplemental material 2—included studies in this systematic review

## CLNM group

| 1 | Chang L, Zhang Y, Zhu J, et al. An integrated nomogram combining deep learning, clinical characteristics and ultrasound features for predicting central lymph node metastasis in papillary thyroid cancer: A multicenter study. *Front Endocrinol (Lausanne).* 2023;14:964074. |
| --- | --- |
| 2 | Chang Q, Zhang J, Wang Y, et al. Nomogram model based on preoperative serum thyroglobulin and clinical characteristics of papillary thyroid carcinoma to predict cervical lymph node metastasis. *Front Endocrinol (Lausanne).* 2022;13:937049. |
| 3 | Chen F, Jiang S, Yao F, et al. A nomogram based on clinicopathological and ultrasound characteristics to predict central neck lymph node metastases in papillary thyroid cancer. *Front Endocrinol (Lausanne).* 2023;14:1267494. |
| 4 | Chen H, Pan WK, Ren SY, Zhou YL. An Online Model for Central Lymph Node Metastases in Papillary Thyroid Carcinoma With BRAF V600E Mutation. *Am J Clin Oncol.* 2024. |
| 5 | Chen Q, Liu Y, Liu J, Su Y, Qian L, Hu X. Development and validation of a dynamic nomogram based on conventional ultrasound and contrast-enhanced ultrasound for stratifying the risk of central lymph node metastasis in papillary thyroid carcinoma preoperatively. *Front Endocrinol (Lausanne).* 2023;14:1186381. |
| 6 | Dai Q, Liu D, Tao Y, et al. Nomograms based on preoperative multimodal ultrasound of papillary thyroid carcinoma for predicting central lymph node metastasis. *Eur Radiol.* 2022;32(7):4596-4608. |
| 7 | Dai Q, Liu D, Tao Y, et al. Nomograms based on preoperative multimodal ultrasound of papillary thyroid carcinoma for predicting central lymph node metastasis. *Eur Radiol.* 2022;32(7):4596-4608. |
| 8 | Deng Y, Zhang J, Wang J, et al. Risk factors and prediction models of lymph node metastasis in papillary thyroid carcinoma based on clinical and imaging characteristics. *Postgrad Med.* 2023;135(2):121-127. |
| 9 | Du J, Yang Q, Sun Y, et al. Risk factors for central lymph node metastasis in patients with papillary thyroid carcinoma: a retrospective study. *Front Endocrinol (Lausanne).* 2023;14:1288527. |
| 10 | Feng JW, Hong LZ, Wang F, et al. A Nomogram Based on Clinical and Ultrasound Characteristics to Predict Central Lymph Node Metastasis of Papillary Thyroid Carcinoma. *Front Endocrinol (Lausanne).* 2021;12:666315. |
| 11 | Feng JW, Liu SQ, Qi GF, et al. Development and Validation of Clinical-Radiomics Nomogram for Preoperative Prediction of Central Lymph Node Metastasis in Papillary Thyroid Carcinoma. *Acad Radiol.* 2024;31(6):2292-2305. |
| 12 | Gao X, Luo W, He L, Cheng J, Yang L. Predictors and a Prediction Model for Central Cervical Lymph Node Metastasis in Papillary Thyroid Carcinoma (cN0). *Frontiers in Endocrinology.* 2022;12. |
| 13 | He L, Chen X, Hu J, et al. Score based on contrast-enhanced ultrasound predict central lymph node metastasis in papillary thyroid cancer. *Front Endocrinol (Lausanne).* 2024;15:1336787. |
| 14 | Hei H, Zhou B, Gong W, Zheng C, Fang J, Qin J. Preoperative prediction of central neck metastasis in patients with clinical T1-2N0 papillary thyroid carcinoma. *Surg Today.* 2023;53(4):507-512. |
| 15 | Hu Q, Zhang WJ, Liang L, et al. Establishing a Predictive Nomogram for Cervical Lymph Node Metastasis in Patients With Papillary Thyroid Carcinoma. *Front Oncol.* 2021;11:766650. |
| 16 | Hu W, Zhuang Y, Tang L, et al. Preoperative Cervical Lymph Node Metastasis Prediction in Papillary Thyroid Carcinoma: A Noninvasive Clinical Multimodal Radiomics (CMR) Nomogram Analysis. *J Oncol.* 2023;2023:3270137. |
| 17 | Huang C, Cong S, Liang T, et al. Development and validation of an ultrasound-based nomogram for preoperative prediction of cervical central lymph node metastasis in papillary thyroid carcinoma. *Gland Surg.* 2020;9(4):956-967. |
| 18 | Huang Y, Huang Z, Cai H, et al. Evaluation of serum B7-H3 expression, ultrasound and clinical characteristics to predict the risk of cervical lymph node metastases in papillary thyroid carcinoma by nomogram. *J Clin Lab Anal.* 2023;37(1):e24811. |
| 19 | Jiang L, Zhang Z, Guo S, Zhao Y, Zhou P. Clinical-Radiomics Nomogram Based on Contrast-Enhanced Ultrasound for Preoperative Prediction of Cervical Lymph Node Metastasis in Papillary Thyroid Carcinoma. *Cancers (Basel).* 2023;15(5). |
| 20 | Li J, Sun P, Huang T, et al. Preoperative prediction of central lymph node metastasis in cN0T1/T2 papillary thyroid carcinoma: A nomogram based on clinical and ultrasound characteristics. *Eur J Surg Oncol.* 2022;48(6):1272-1279. |
| 21 | Lin P, Liang F, Ruan J, et al. A Preoperative Nomogram for the Prediction of High-Volume Central Lymph Node Metastasis in Papillary Thyroid Carcinoma. *Front Endocrinol (Lausanne).* 2021;12:753678. |
| 22 | Liu W, Zhang D, Jiang H, et al. Prediction model of cervical lymph node metastasis based on clinicopathological characteristics of papillary thyroid carcinoma: a dual-center retrospective study. *Front Endocrinol (Lausanne).* 2023;14:1233929. |
| 23 | Pang J, Yang M, Li J, et al. Interpretable machine learning model based on the systemic inflammation response index and ultrasound features can predict central lymph node metastasis in cN0T1-T2 papillary thyroid carcinoma. *Gland Surg.* 2023;12(11):1485-1499. |
| 24 | Qiao D, Deng X, Liang R, et al. Nomogram to predict central lymph node metastasis in papillary thyroid carcinoma. *Clin Exp Metastasis.* 2024. |
| 25 | Song X, Skog S, Wei L, et al. Nomogram model of serum thymidine kinase 1 combined with ultrasonography for prediction of central lymph node metastasis risk in patients with papillary thyroid carcinoma pre-surgery. *Front Endocrinol (Lausanne).* 2024;15:1366219. |
| 26 | Sun F, Zou Y, Huang L, et al. Nomogram to Assess the Risk of Central Cervical Lymph Node Metastasis in Patients With Clinical N0 Papillary Thyroid Carcinoma. *Endocr Pract.* 2021;27(12):1175-1182. |
| 27 | Wang Z, Chang Q, Zhang H, et al. A Clinical Predictive Model of Central Lymph Node Metastases in Papillary Thyroid Carcinoma. *Front Endocrinol (Lausanne).* 2022;13:856278. |
| 28 | Wei L, Wu Y, Bo J, et al. Dual-Energy Computed Tomography Parameters Combined With Inflammatory Indicators Predict Cervical Lymph Node Metastasis in Papillary Thyroid Cancer. *Cancer Control.* 2024;31:10732748241262177. |
| 29 | Wen Q, Wang Z, Traverso A, et al. A radiomics nomogram for the ultrasound-based evaluation of central cervical lymph node metastasis in papillary thyroid carcinoma. *Front Endocrinol (Lausanne).* 2022;13:1064434. |
| 30 | Xue J, Li S, Qu N, et al. Value of clinical features combined with multimodal ultrasound in predicting lymph node metastasis in cervical central area of papillary thyroid carcinoma. *Journal of Clinical Ultrasound.* 2023;51(5):908-918. |
| 31 | Xue T, Liu C, Liu JJ, et al. Analysis of the Relevance of the Ultrasonographic Features of Papillary Thyroid Carcinoma and Cervical Lymph Node Metastasis on Conventional and Contrast-Enhanced Ultrasonography. *Front Oncol.* 2021;11:794399. |
| 32 | Yang Z, Heng Y, Lin J, et al. Nomogram for Predicting Central Lymph Node Metastasis in Papillary Thyroid Cancer: A Retrospective Cohort Study of Two Clinical Centers. *Cancer Res Treat.* 2020;52(4):1010-1018. |
| 33 | Zeng B, Min Y, Feng Y, Xiang K, Chen H, Lin Z. Hashimoto's Thyroiditis Is Associated With Central Lymph Node Metastasis in Classical Papillary Thyroid Cancer: Analysis from a High-Volume Single-Center Experience. *Front Endocrinol (Lausanne).* 2022;13:868606. |
| 34 | Zhang H, Hu S, Wang X, et al. Using Diffusion-Weighted MRI to Predict Central Lymph Node Metastasis in Papillary Thyroid Carcinoma: A Feasibility Study. *Front Endocrinol (Lausanne).* 2020;11:326. |
| 35 | Zhou SC, Liu TT, Zhou J, et al. An Ultrasound Radiomics Nomogram for Preoperative Prediction of Central Neck Lymph Node Metastasis in Papillary Thyroid Carcinoma. *Front Oncol.* 2020;10:1591. |
| 36 | Tong Y, Zhang J, Wei Y, et al. Ultrasound-based radiomics analysis for preoperative prediction of central and lateral cervical lymph node metastasis in papillary thyroid carcinoma: a multi-institutional study. *BMC Med Imaging.* 2022;22(1):82. |
| 37 | Zhao D, Li W, Zhang X. Development and validation of a nomogram for preoperative prediction of ipsilateral cervical central lymph node metastasis in papillary thyroid cancer: a population-based study. *Gland Surg.* 2024;13(4):528-539. |

## LLNM group

| 1 | Chang Q, Zhang J, Wang Y, et al. Nomogram model based on preoperative serum thyroglobulin and clinical characteristics of papillary thyroid carcinoma to predict cervical lymph node metastasis. *Front Endocrinol (Lausanne).* 2022;13:937049. |
| --- | --- |
| 2 | Dong L, Han X, Yu P, et al. CT Radiomics-Based Nomogram for Predicting the Lateral Neck Lymph Node Metastasis in Papillary Thyroid Carcinoma: A Prospective Multicenter Study. *Acad Radiol.* 2023;30(12):3032-3046. |
| 3 | Dou Y, Chen Y, Hu D, Xiong W, Xiao Q, Su X. Development and validation of web-based nomograms for predicting lateral lymph node metastasis in patients with papillary thyroid carcinoma. *Gland Surg.* 2020;9(2):172-182. |
| 4 | Dou Y, Chen Y, Hu D, Xiong W, Xiao Q, Su X. Development and validation of web-based nomograms for predicting lateral lymph node metastasis in patients with papillary thyroid carcinoma. *Gland Surg.* 2020;9(2):172-182. |
| 5 | Feng JW, Wu WX, Qi GF, et al. Nomograms based on sonographic and clinicopathological characteristics to predict lateral lymph node metastasis in classic papillary thyroid carcinoma. *J Endocrinol Invest.* 2022;45(11):2043-2057. |
| 6 | Feng JW, Wu WX, Qi GF, et al. Nomograms based on sonographic and clinicopathological characteristics to predict lateral lymph node metastasis in classic papillary thyroid carcinoma. *J Endocrinol Invest.* 2022;45(11):2043-2057. |
| 7 | Feng JW, Ye J, Hong LZ, et al. Nomograms for the prediction of lateral lymph node metastasis in papillary thyroid carcinoma: Stratification by size. *Front Oncol.* 2022;12:944414. |
| 8 | Heng Y, Yang Z, Zhou L, Lin J, Cai W, Tao L. Risk stratification for lateral involvement in papillary thyroid carcinoma patients with central lymph node metastasis. *Endocrine.* 2020;68(2):320-328. |
| 9 | Liu S, Liu C, Zhao L, et al. A prediction model incorporating the BRAF(V600E) protein status for determining the risk of cervical lateral lymph node metastasis in papillary thyroid cancer patients with central lymph node metastasis. *Eur J Surg Oncol.* 2021;47(11):2774-2780. |
| 10 | Liu W, Zhang D, Jiang H, et al. Prediction model of cervical lymph node metastasis based on clinicopathological characteristics of papillary thyroid carcinoma: a dual-center retrospective study. *Front Endocrinol (Lausanne).* 2023;14:1233929. |
| 11 | Ma Y, Li Y, Zheng L, He Q. Prospective application of a prediction model for lateral lymph node metastasis in papillary thyroid cancer patients with central lymph node metastasis. *Frontiers in Endocrinology.* 2024;14. |
| 12 | Tong Y, Li J, Huang Y, et al. Ultrasound-Based Radiomic Nomogram for Predicting Lateral Cervical Lymph Node Metastasis in Papillary Thyroid Carcinoma. *Acad Radiol.* 2021;28(12):1675-1684. |
| 13 | Tong Y, Zhang J, Wei Y, et al. Ultrasound-based radiomics analysis for preoperative prediction of central and lateral cervical lymph node metastasis in papillary thyroid carcinoma: a multi-institutional study. *BMC Med Imaging.* 2022;22(1):82. |
| 14 | Wang J, Gao Y, Zong Y, et al. Nomogram Model Based on Iodine Nutrition and Clinical Characteristics of Papillary Thyroid Carcinoma to Predict Lateral Lymph Node Metastasis. *Cancer Control.* 2023;30:10732748231193248. |
| 15 | Zhao L, Zhou T, Zhang W, et al. Blood immune indexes can predict lateral lymph node metastasis of thyroid papillary carcinoma. *Front Endocrinol (Lausanne).* 2022;13:995630. |
| 16 | Zhu J, Chang L, Li D, et al. Nomogram for preoperative estimation risk of lateral cervical lymph node metastasis in papillary thyroid carcinoma: a multicenter study. *Cancer Imaging.* 2023;23(1):55. |
| 17 | Zhuo X, Yu J, Chen Z, et al. Dynamic Nomogram for Predicting Lateral Cervical Lymph Node Metastasis in Papillary Thyroid Carcinoma. *Otolaryngol Head Neck Surg.* 2022;166(3):444-453. |
| 18 | Zou Y, Sun S, Liu Q, et al. A new prediction model for lateral cervical lymph node metastasis in patients with papillary thyroid carcinoma: Based on dual-energy CT. *Eur J Radiol.* 2021;145:110060. |
| 19 | Gong J, Zhu B, Liu W, et al. Risk Factors for Lymph Node Metastasis in Papillary Thyroid Carcinoma: A Retrospective Study. *Horm Metab Res.* 2023;55(5):315-322. |
| 20 | Huang C, Hu D, Zhuang Y, Su X. Risk factors and prediction model of level II lymph node metastasis in papillary thyroid carcinoma. *Frontiers in Oncology.* 2022;12. |

# Supplemental material 3—China heatmap of predictive model of PTC-LNM


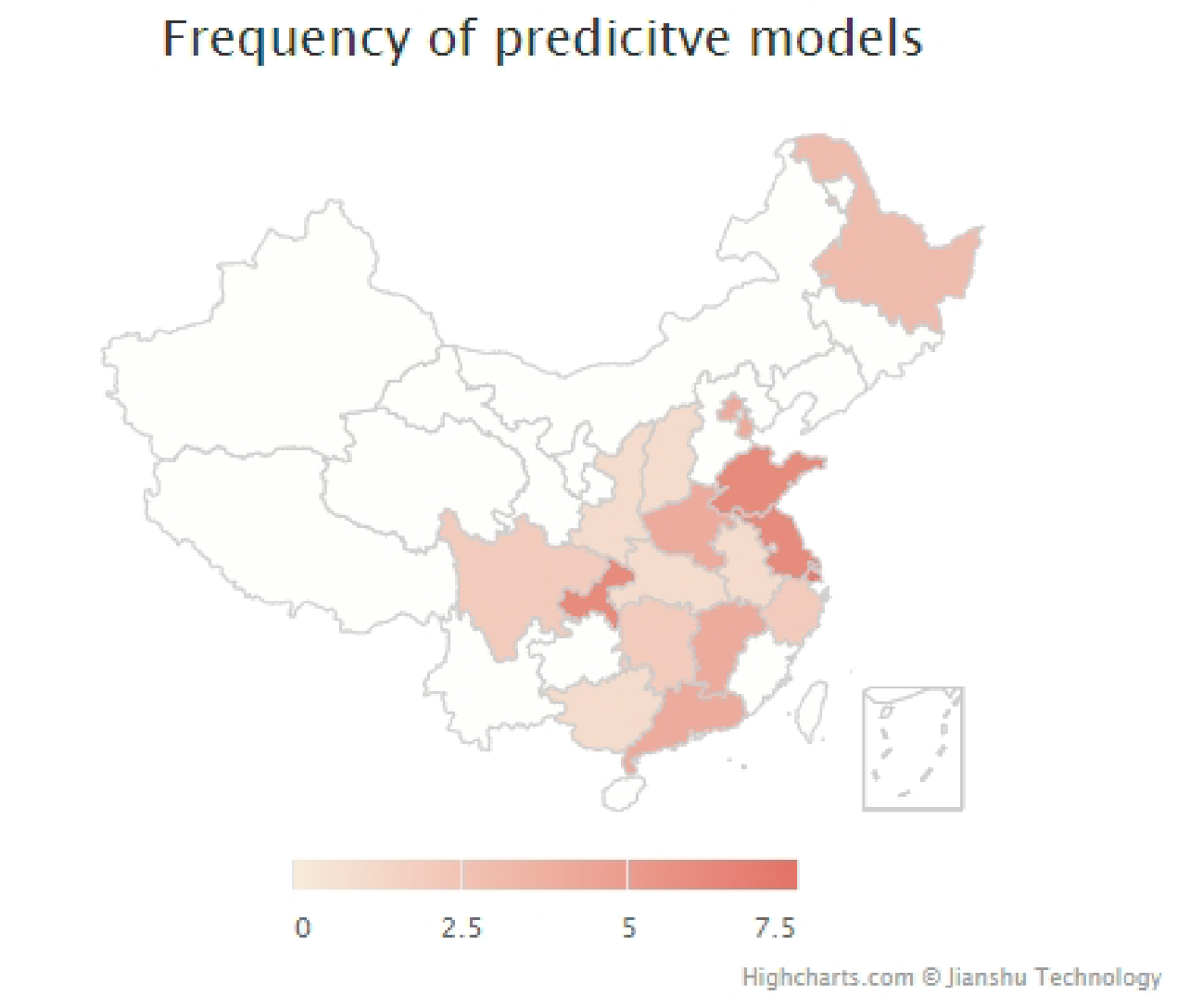


A

A, Frequency of developing predictive models


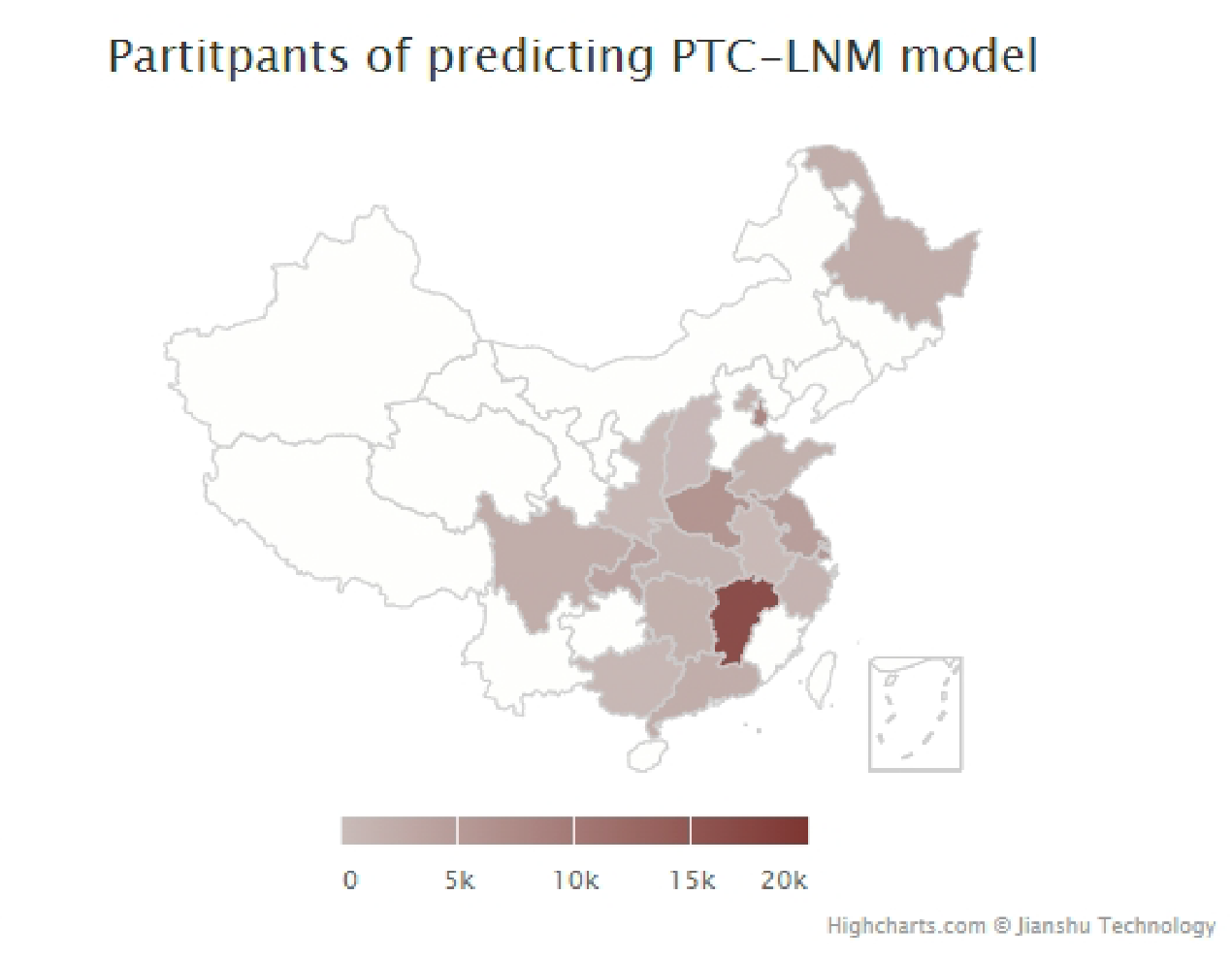


B

B, Number of participants in the development of predictive models.

# Supplemental material 4—PROBAST risk of bias for external validation

| **Validation Paper** | **Outcome** | **EX-VALIDATION STUDY** | | | | | | | | |
| --- | --- | --- | --- | --- | --- | --- | --- | --- | --- | --- |
|  |  | **RISK OF BIAS (- / ? / +)** | | | | **APPLICABILITY (- / ? / +)** | | | **OVERALL (- / ? / +)** | |
|  |  | **Participants** | **Predictors** | **Outcome** | **Analysis** | **Participants** | **Predictors** | **Outcome** | **RoB** | **Applicability** |
| Chang, L | CLNM | + | + | + | - | + | + | + | - | + |
| Chang, Q | CLCM | + | + | + | + | + | + | + | + | + |
| Liu, W | CLNM | + | + | + | ? | + | + | + | ? | + |
| Sun, F | CLNM | + | + | + | + | + | + | + | + | + |
| Tong, Y | CLNM | + | + | ? | - | + | + | + | - | + |
| Chang, Q | LLCM | + | + | + | - | + | + | + | - | + |
| Dong, L | LLCM | + | + | + | - | + | + | + | - | + |
| Liu, W | LLNM | + | + | + | ? | + | + | + | ? | + |
| Tong, Y | LLNM | + | + | ? | - | + | + | + | - | + |
| Zhao, L | LLNM | + | + | + | - | + | + | + | - | + |
| Zhu, J | LLNM | + | + | + | + | + | + | + | + | + |
| Zou, Y | LLNM | + | + | + | - | + | + | + | - | + |

The overall ROB (25%) and applicability (100%) with external validation demonstrated low risk, which may be related to suitable methods.

Supplemental material 4—TRIPOD+AI Radar Chart for all external validation study (CLNM outcome: A-E; LLNM outcome: F-L).


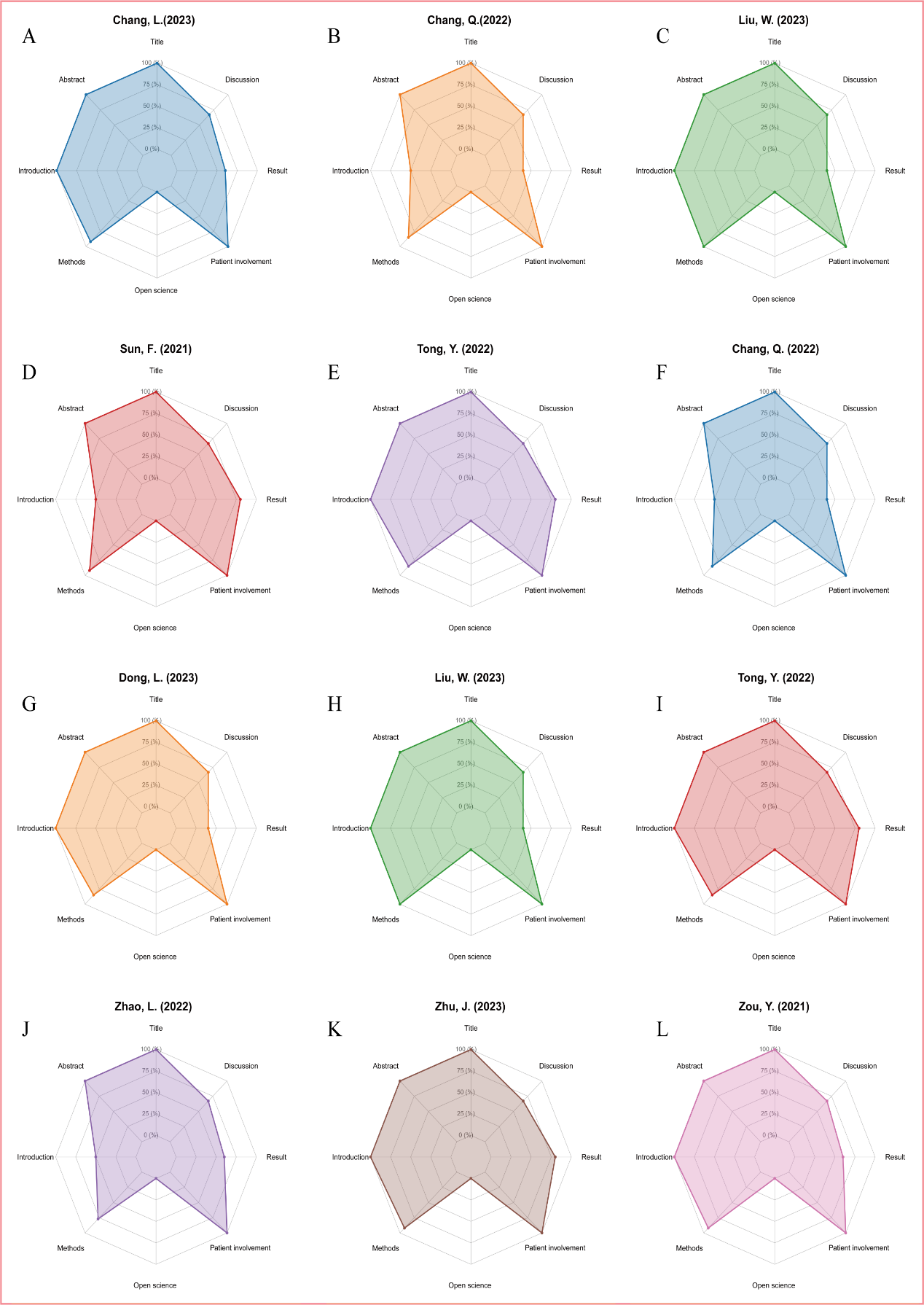


# Supplemental material 5—Frequency of risk factors

| CLNM-group | | |
| --- | --- | --- |
|  |  | Frequency |
| Included factors |  | 4~22 |
| Risk factors |  | 3~10 |
|  | Tumor size | 27 |
|  | Age | 26 |
|  | Gender | 19 |
|  | Multifocality | 15 |
|  | Capsular invasion (&ETE) | 14 |
|  | Tumor position | 7 |
|  | BRAF muation | 4 |
|  |  |  |
| Feature categories | Ultrasound | 35 |
|  | Radiomics | 6 |
|  | Laboratory indicators | 9 |

| LLNM-group | | |
| --- | --- | --- |
|  |  | Frequency |
| Included factors |  | 4~14 |
| Risk factors |  | 2~11 |
|  | Tumor size | 15 |
|  | CLNM (or suspicious +) | 12 |
|  | Tumor position | 8 |
|  | Multifocality | 8 |
|  | Age | 7 |
|  | Capsular invasion (&ETE) | 5 |
|  | Gender | 4 |
|  |  |  |
| Feature categories | Ultrasound | 19 |
|  | Radiomics | 6 |
|  | Laboratory indicators | 3 |

# Supplemental material 6—Risk factors of nomogram

## Table 1 Risk variables of CLNM group nomograms

| **Author** | **Year** | **Outcome** | **Feature categories** | **Variables included in screening** | **No. of factors** | **Risk factors of nomogram** | **No. of risk factors** |
| --- | --- | --- | --- | --- | --- | --- | --- |
| Chang, L | 2023 | CLNM | C/U | gender, age≤55, tumor size, tumor position, multifocality, taller-than-wide, echo, microcalcification, A/P>0.25, US-reported CLN status, AI model-predicted value | 11 | AI model-predicted value, multifocality, tumor position, microcalcification, A/P>0.25, US-reported CLN status | 6 |
| Chang, Q | 2022 | CLNM | C/U/B | gender, age<45, PS-Tg level ≥31.650, ETE, maximum tumor size >1 cm, multifocality positive | 6 | age<45, gender, maximum tumor size >1 cm, PS-Tg ≥31.650 ng/ml, ETE, multifocality | 6 |
| Chen, F | 2023 | CLNM | C/U | age＞55, gender, CN0, tumor size≥1cm, BRAFV600E mutation, multifocality, bilateral tumor, extrathyroidal extension, ACR scores | 9 | gender, age >55 years, clinical lymph node positivity, tumor size ≥1 cm, ACR scores ≥6 | 5 |
| Chen, H | 2024 | CLNM | C/U | Age (continuous variable), gender, tumor size (continuous variable), multifocality, capsular invasion, pathological subtypes | 6 | gender, age(continuous variable), pathological subtype, multifocality, tumor size(continuous variable) | 5 |
| Chen, Q | 2023 | CLNM | C/U | gender, age≤ 42 years, size≥0.95mm, peak intensity, Degree of homogeneity | 5 | gender, age≤ 42 years, size, peak intensity, Degree of homogeneity | 5 |
| Dai, Q | 2022 | CLNM | C/U | Gender, Age<45, Tumor size＞10mm, Quantity, Position, Orientation, Location, Multifocality, Shape, Margin, Boundary, Echo pattern, Echo pattern, Aspect ratio＞1, Posterior features, Calcification, Microcalcification pattern, Capsule protrusion, Capsular relation, Composition, C-TIRADS, Alder grade, Elastography score, Thyroid background, Preoperative US of LN, Found suspicious CLN, LLN-aspect ratio≥2, LLN-shape, LLN-central hilum, LLN-echo, LLN-microcalcification, LLN-necrosis, LLN-CDFI | 22 | age, multifocality, location, capsular relation, echo texture, Alder grade, elastography score | 7 |
| Dai, Q | 2022 | CLNM | C/U | Gender, Age≥45, Tumor size ＞10mm, Quantity, Position, Orientation, Location, Multifocality, Shape, Margin, Boundary, Echo pattern, Echo pattern, Aspect ratio＞1, Posterior features, Calcification, Microcalcification pattern, Capsule protrusion, Capsular relation, Composition, C-TIRADS, Alder grade, Elastography score, Thyroid background, Preoperative US of LN, Found suspicious CLN, LLN-aspect ratio≥2, LLN-shape, LLN-central hilum, LLN-echo, LLN-microcalcification, LLN-necrosis, LLN-CDFI | 22 | Age<45, multifocality, location, capsular relation, Alder grade, elastography score, found suspicious CLN, LLN CDFI | 8 |
| Deng, Y | 2023 | CLNM | C/U | Age (continuous variable), gender, body weight, systolic blood pressure, free triiodothyronine, nodule location, nodule number, TI-RADS grade, imaging logical, nodule diameter(continuous variable) | 10 | gender, age (continuous variable), imaging logical, nodule diameter (continuous variable), TI-RADS grade | 5 |
| Du, J | 2023 | CLNM | C/U | age≤45 years, BMI ≥25, tumor size ≥1 cm, capsular invasion, BRAF V600E mutation | 5 | age≤45 years, BMI ≥25, size ≥1 cm, capsular invasion, BRAF V600E mutation | 5 |
| Feng, J. W | 2021 | CLNM | C/U | gender, CLT, tumor size>1cm, multifocality, the number of foci, tumor location, A/T, margin, echogenic foci | 9 | gender, chronic lymphocytic thyroiditis, tumor size>1cm, the number of foci, tumor location, margin | 6 |
| Feng, J. W | 2024 | CLNM | C/U/R | gender, Age<55, CLT, Tumor size>1cm, The number of foci, Multifocality, Margin, Echogenic foci, LLNM, Radiomics signature, Serum TG | 11 | age< 55, tumor size>1cm, margin, lateral lymph node metastasis, radiomics signature | 5 |
| Gao, X | 2021 | CLNM | C/U/B | age≤40 years, tumor size>1.0 cm, capsule contact, microcalcifications, blood flow signal (poor), TgAb (positive), BRAFV600E status mutation | 7 | TgAb (positive), microcalcifications, tumor size >1.0 cm | 3 |
| He, L | 2024 | CLNM | C/U | shape, tumor size ≥ 1.0cm, longest diameter, anteroposterior diameter, margin, Echogenic foci, enhancement direction, peak intensity | 8 | shape, CEUS TI-RADS, tumor size ≥ 1.0cm | 3 |
| Hei, H | 2023 | CLNM | C/U | gender, age (continuous variable), tumor size (continuous variable), T classification | 4 | gender, age (continuous variable), tumor size (continuous variable) | 3 |
| Hu, Q | 2021 | CLNM | C/U | gender, Age < 45 years, expression of galectin-3, Tumor size >20mm, boundary, margin, bilaterality, taller than wide, multifocality, tumor position, echogenicity, presence of microcalcification, extracapsular invasion, US suggested CLNM | 14 | gender, Age < 45 years, Tumor size >20mm, multifocality, ambiguous boundary, extracapsular invasion, US-suggested lymph nodes metastasis | 7 |
| Hu, W | 2023 | CLNM | C/U/R | Diameter<1 cm, Location, Tumor margin on T1WC+, Thyroid contour protrusion sign on T1WC+, Aspect ratio on US imaging, Microcalcification, MOGONET | 7 | poorly defined tumor maigin, thyroid contour protrusion, MOGONET | 3 |
| Huang, C | 2020 | CLNM | C/U | gender, age (continuous variable), tumor size (continuous variable), multiple tumors, US-based CLNM status | 5 | gender, age (continuous variable), tumor size (continuous variable), multiple tumors, US-based CLNM status | 5 |
| Huang, Y | 2023 | CLNM | C/B | gender, age＜55, tumor size≥1cm, Multifocal, High sB7-H3 expression | 5 | gender, age＜55, tumor size≥1cm, Multifocal, High sB7-H3 expression | 5 |
| Jiang, L | 2023 | CLNM | C/U | age<55, gender, tumor size>10 mm, US-reported LN status, BMUS Radscore, CEUS Radscore | 6 | Gender, age < 55, US-reported LN status positive, CEUS Radscore | 4 |
| Li, J | 2022 | CLNM | C/U | gender, age≤55, maximum diameter>1cm, multifocality, capsular invasion, infiltrative margin, irregular shape, calcification, intra-nodular vascularity, A/T>1 | 10 | gender, age≤55, maximum diameter>1cm, multifocality, capsular invasion, infiltrative margins, intra-nodular vascularity, A/T >1 | 8 |
| Lin, P | 2021 | CLNM | C/U | age≤35, BRAF V600E mutation, nodule diameter (continuous variable), calcification, Gender, Hashimoto’s thyroiditis, Multiple Suspicious Malignant Foci, TIRADS of 4a/4b/4c/5, Solid composition, A/T > 1, Margin, Vascularity Pattern, intranodular | 13 | age≤35, BRAF V600E mutation, nodule diameter (continuous variable), calcification | 4 |
| Liu, W | 2023 | CLNM | C/B | Age<45, gender, tumor extension, tumor size>1cm, tumor location, serum FT3, multifocality, serum TSH, FT3/FT4 | 9 | age <45, gender, tumor diameter > 1 cm, tumor location, mETE, serum TSH ≥1.418 | 6 |
| Pang, J | 2023 | CLNM | C/U/B | gender, Age<55, Tumor size<10mm, Capsular invasion, Laterality, Multifocality, Location, Solid composition, Unclear margin, Microcalcifications, CDFI blood flow, Hashimoto thyroiditis, NLR, MLR, SIRI | 15 | gender, Age<55, Tumor size<10mm, Capsular invasion, Location, Unclear margin, Microcalcifications, CDFI blood flow, Hashimoto thyroiditis, SIRI | 10 |
| Qiao, D | 2024 | CLNM | C/U/B | HT, calcification, multifoci, capsular invasion, PLR > 130.34, larger tumor diameter (continuous variable), position | 7 | HT, calcification, multifoci, capsular invasion, PLR > 130.34, larger tumor diameter (continuous variable), position | 7 |
| Song, X | 2024 | CLNM | C/U/B | echogenic foci, margin, tumor number ≥2, maximum tumor size>1cm, capsule invasion, CLN flow signal, STK1p pre-surgery | 7 | maximum tumor size ≥1 cm, capsule invasion, irregular margin, CLN flow signal, tumor-foci number ≥2, STK1p ≥1.7 pmol/L | 6 |
| Sun, F | 2021 | CLNM | C/U | gender, diameter (continuous variable), location, margin, shape, calcification, A/P | 7 | Diameter (continuous variable), shape, calcification, A/P | 4 |
| Wang, Z | 2022 | CLNM | C/U | Age, Gender, Multifocality, BRAF, Tumor size≥1cm, FT3, FT4, TSH, TPO, TgAb, Tg, TERT | 12 | Age, Gender, Multifocality, BRAF, Tumor size≥ 1 cm | 5 |
| Wei, L | 2024 | CLNM | C/U/R | age<45, gender, calcification, tumor diameter<10mm, IC, NIC, λ HU, dNLR, PNI | 9 | gender, tumor diameter<10mm, NIC, dNLR, PNI | 5 |
| Wen, Q | 2022 | CLNM | C/U/R | age<45, gender, BRAF V600E mutation status, tumor size≥10mm, tumor internal echo pattern, ETE, radiomics signature | 7 | age<45, gender, BRAF V600E mutation status, radiomics signature, ETE | 5 |
| Xue, J | 2023 | CLNM | C/U/R | gender, Number of lesions, Microcalcification, capsule invasion, Reinforcement strength, multimodal ultrasound Rad-score | 6 | gender, Number of lesions, capsule invasion, Reinforcement strength, multimodal ultrasound Rad-score | 5 |
| Xue, T | 2021 | CLNM | C/U | gender, age≤45, tumor size≥1cm, microcalcification, contact extent, multifocality, capsule integrity, enhancement patterns in CEUS | 8 | tumor size≥1cm, age ≤45, multifocality, contact extent >50% | 4 |
| Yang, Z | 2020 | CLNM | C/U/B | bilaterality, CEA, Cr, RBC, multifocality, maximum tumor diameter≥1cm, TCI, Hashimoto thyroiditis, BMI, age≤40 | 10 | thyroid capsular invasion, multifocality, Cr> 70 μmol/L, age < 40, tumor size > 1 cm, BMI < 22, CEA> 1 ng/mL | 7 |
| Zeng, B | 2022 | CLNM | C/U | gender, age<55, tumor size>1cm, presence of HT condition | 4 | gender, age<55, tumor size>1cm, presence of HT condition | 4 |
| Zhang, H | 2020 | CLNM | C/U | gender, tumor size≥1.65cm, multifocality, bilaterality, ETE, ADC500 | 6 | gender, tumor size≥1.65cm, ETE, ADC500 Value | 4 |
| Zhou, S. C | 2020 | CLNM | C/U/B | Gender, US reported LN status, Bethesda category, TSH, TG, TGAB, TPOAB, US radiomics signature | 8 | age, TG level, TPOAB level, US reported LN status, US radiomics signature | 5 |
| Tong, Y | 2022 | CLNM | C/U/R | * | * | radiomics, age≤ 45, US-reported central CLN status | 3 |
| Zhao, D | 2024 | CLNM | C/U | Age (continuous variable), history of osteoporosis, complicated by HT, TSH level, TPOAb, eGFR level, tumor size>1 cm, nodule boundary, nodule shape, microcalcification, ELN, ETE | 12 | Age (continuous variable), history of osteoporosis, complicated by HT, ELN, ETE | 5 |

C, clinical; U, ultrasound; R, radiology；B=biochemical; P, mutation and genes.

## Table 2 Risk variables of LLNM group nomograms

| **Author** | **Year** | **Outcome** | Feature categories | **Variables included in screening** | **No. of factors** | **Risk factors of nomogram** | **No. of risk factors** |
| --- | --- | --- | --- | --- | --- | --- | --- |
| Chang, Q | 2022 | LLNM | C/U/B | gender, age<45, maximum tumor size>1 cm, PS-Tg ≥30.175 ng/ml, CLNM positive, ETE, multifocality | 7 | maximum tumor size>1 cm, PS-Tg ≥30.175 ng/ml, CLNM positive, ETE, multifocality | 5 |
| Dong, L | 2023 | LLNM | C/R | gender, Age (continuous variable), TSH, Tumor diameter, Tumor location, Hashimoto antibody, Number of suspected tumors, Rad-score | 8 | Age (continuous variable), Tumor diameter, Tumor location, Number of suspected tumors, Rad-score | 5 |
| Dou, Y | 2020 | LLNM/II | C/U | tumor size≥1cm, tumor location, ETE, tumor bilaterality, Prelaryngeal, Pretracheal, Paratracheal | 7 | tumor size≥1cm, tumor location, Prelaryngeal, Paratracheal | 4 |
| Dou, Y | 2020 | LLNM/III+IV | C/U | tumor size≥1cm, tumor location, ETE, tumor bilaterality, Prelaryngeal, Pretracheal, Paratracheal | 7 | tumor size≥1cm, tumor location, Prelaryngeal, Pretracheal, Paratracheal | 5 |
| Feng, J. W | 2022 | LLNM | C/U | age≥ 55, tumor size>1cm, the number of foci, tumor location, A/T, margin, ETE, echogenic foci, CLNM, LNR, Number of metastatic LNs in central compartment≥5 | 11 | tumor size, the number of foci, tumor location, ETE, CLNM, LNR, | 6 |
| Feng, J. W | 2022 | mLLNM | C/U | age ≥ 55, BMI, margin, ETE, the number of metastatic lymph nodes in the central compartment ≥ 6 | 5 | age ≥ 55, ETE, the number of metastatic lymph nodes in the central compartment ≥ 6 | 3 |
| Feng, J. W | 2022 | LLNM | C/U | NI | NI | CLT, margin, location, number, CLNM, CLNR | 6 |
| Heng, Y | 2020 | LLNM | C/U | age ≤ 40, maximum tumor diameter ≥ 1.0 cm, TCI, ipsilateral nodular goiter | 4 | age ≤ 40, maximum tumor diameter ≥ 1.0 cm, TCI, ipsilateral nodular goiter | 4 |
| Liu, S | 2021 | LLNM | C/U | gender, Tumor Size≥1cm, multifocality, BRAFV600E status, CLNM | 5 | BRAFV600E status, multifocality, Tumor Size≥1cm, CLNM | 4 |
| Liu, W | 2023 | LLNM | C/U/B | age<45, gender, tumor extension, tumor size>1cm, tumor location, multifocality, serum TSH, serum FT3, CLNM | 9 | age <45, tumor size>1cm, extension, multifocality, serum TSH ≥2.910, CLNM | 6 |
| Ma, Y | 2023 | LLNM | C/U | gender, maximum tumor diameter> 1.0 cm, BRAFV600E mutation, multifocality, the number of CLNM ≥3, the ratio of CLNM ≥0.297, prelaryngeal LNM | 7 | maximum tumor diameter> 1.0 cm, multifocality, the number of CLNM ≥3, the ratio of CLNM ≥0.297, tumor location | 5 |
| Tong, Y | 2021 | LLNM | C/U/R | Tumor size (continuous variable), Tumor size≥1cm, US radiomics signature, US-reported CLN status, CT-reported CLN status, | 5 | US radiomics signature, US-reported CLN status, CT-reported CLN status | 3 |
| Tong, Y | 2022 | LLNM | C/U/R | NI | NI | radiomics signature, US-reported lateral CLN status | 2 |
| Wang, J | 2023 | LLNM | C/U/B | Age (continuous variable), gender, SIC>45Ug/L, smoking, drinking, family history, multifocality, unilateral or bilateral, TSH (continuous variable), Tg (continuous variable), size>2cm | 11 | Age (continuous variable), gender, SIC>45Ug/L, smoking, drinking, family history, multifocality, unilateral or bilateral, TSH (continuous variable), Tg (continuous variable), size>2cm | 11 |
| Zhao, L | 2022 | LLNM | C/U/B | Age (continuous variable), tumor diameter (continuous variable), SII(continuous variable), bilateral tumor, multifocality | 5 | tumor diameter (continuous variable), SII (continuous variable) | 2 |
| Zhu, J | 2023 | LLNM | C/U | age≤ 55, gender, tumor size>1cm, tumor position, internal echo, microcalcification, vascularization, A/T>0.25, mulifocality | 9 | tumor position, gender, microcalcification, tumor size, mulifocality, A/P > 0.25 | 6 |
| Zhuo, X | 2022 | LLNM | C/U/R | age<55, gender, tumor size≥10.5mm, thyroid nodules, ETE, irregular tumor shape, tumor boundary, tumor echo, capsular invasion, tumor calcification, tumor vascularity, ultrasound features of lymph nodes (location, smallest diameter, maximum diameter, calcification, vascularity, vascularity type), computed tomography features of lymph nodes (diameter, enhancement, uneven enhancement, cystic changes, calcification) | 14 | gender, tumor size ≥10.5 mm, irregular tumor shape, thyroid nodules, lymph node location, lymph node vascularity | 6 |
| Zou, Y | 2021 | LLNM | C/R | Age ≤ 55, diameter (continuous variable), IC in the arterial phase, IC in the venous phase, located in superior pole, ETE | 6 | IC in the arterial phase, IC in venous phase, located in superior pole, ETE | 4 |
| Gong, J | 2023 | LLNM | C/U | age<55, gender, lesions, tumor≥1cm, No.CLNM≥3, FPG, ETE | 7 | age<55, gender, lesions, tumor≥1cm, No.CLNM≥3 | 5 |
| Huang, C | 2022 | LLNM/II | C/U | gender, age＜45, expression of galectin-3, tumor maximum diameter > 2cm, boundary, margin, bilaterality, taller than wide, multifocality, tumor position, echogenicity, presence of microcalcification, extracapsular invasion, US suggested CLNM | 13 | tumor maximum diameter > 2cm, location, extracapsular invasion, US suggested CLNM,Rate of CLNM,Rate of LN-level III metastasis,Rate of LN-level IV metastasis | 7 |

C, clinical; U, ultrasound; R, radiology；B=biochemical; P, mutation and genes.

# Supplemental material 7—AUC of included model

## Table 1 Summarize the performance and calibration of the CLNM-group nomograms

| Author | year | Total sample enrolled | Total LNM rate | Statistical model | Validation method | Sample size (TRD) | LNM rate (TrD) | Sample size (INV) | LNM rate (InV) | Sample size (EXV) | LNM rate (ExV) | Feature categories | TRS (95%CI) | INT VAL (95%CI) | EXT VAL (95%CI) | Calibration plot | DCA |
| --- | --- | --- | --- | --- | --- | --- | --- | --- | --- | --- | --- | --- | --- | --- | --- | --- | --- |
| Chang, L | 2023 | 3359 | 1685/3359 | logistic regression | split-sample V (7:3) +EXV | 2114 | 1063/2114 | 906 | 460/906 | 339 | 162/339 | C/U | 0.812 (0.794-0.830) | 0.809 (0.780-0.837) | 0.829 (0.785-0.872) | Yes | Yes |
| Chang, Q | 2022 | 1324 | 461/1324 | logistic regression | split-sample V (3:1) +EXV | 993 | 342/993 | 331 | 119/331 | 631 | * | C/U/B | 0.801 | 0.74 | 0.756 | Yes | Yes |
| Chen, F | 2023 | 691 | 377/691 | logistic regression | bootstrap resampling | 691 | 377/691 | 691 | 377/691 | * |  | C/U | 0.717 | 0.716/0.726 | * | Yes | Yes |
| Chen, H | 2024 | 466 | 323/466 | logistic regression | split-sample V (7:3) | 326 | 228/326 | 140 | 95/140 | * |  | C/U | 0.772 (0.720-0.825) | 0.731 (0.639-0.823) | * | Yes | Yes |
| Chen, Q | 2023 | 216 | 101/216 | LASSO regression + logistic regression | split-sample V (5:5) | 108 | 48/108 | 108 | 53/108 | * |  | C/U | 0.844 (0.755-0.905) | 0.827 (0.747-0.906) | * | Yes | Yes |
| Dai, Q | 2022 | 822 | 399/822 | logistic regression | split-sample V (7:3) | 575 | 279/575 | 247 | 120/247 | * |  | C/U | 0.786 | 0.806 | * | Yes | Yes |
| Dai, Q | 2022 | 822 | 399/822 | logistic regression | split-sample V (7:3) | 575 | 279/575 | 247 | 120/247 | * |  | C/U | 0.789 | 0.804 | * | Yes | Yes |
| Deng, Y | 2023 | 989 | 453/989 | logistic regression | split-sample V (8:2) | 791 | * | 198 | * | * |  | C/U | 0.742 | 0.765 | * | Yes | No |
| Du, J | 2023 | 400 | 140/400 | logistic regression | split-sample V (7:3) | 280 | 98/280 | 120 | 42/120 | * |  | C/U/M | 0.791 (0.735–0.846) | 0.765 (0.677–0.852) | * | Yes | Yes |
| Feng, J. W | 2021 | 886 | 437/866 | logistic regression | split-sample V (7:3) | 617 | 306/617 | 269 | 131/269 | * |  | C/U | 0.806 (0.771 - 0.825) | 0.799 (0.778-0.813) | * | Yes | No |
| Feng, J. W | 2024 | 1069 | 506/1069 | logistic regression | split-sample V (7:3) | 748 | 349/748 | 321 | 157/321 | * |  | C/U | 0.960 (0.947-0.972) | 0.925 (0.895-0.955) | * | Yes | Yes |
| Gao, X | 2021 | 296 | 112/296 | logistic regression | Randomization | 296 | 112/296 | 100/95 | * | * |  | C/U/B | 0.715 | 0.718/0.738 | * | Yes | Yes |
| He, L | 2024 | 684 | 311/684 | logistic regression | split-sample by admission | 495 | 191/495 | 189 | 120/189 | * |  | C/U | 0.72 (0.68- 0.77) | 0.79 (0.72- 0.85) | * | Yes | Yes |
| Hei, H | 2023 | 619 | 169/619 | logistic regression | split-sample by admission | 436 | 111/436 | 183 | 58/183 | * |  | C/P | 0.655 (0.596–0.715 | 0.690 (0.611–0.769) | * | Yes | No |
| Hu, Q | 2021 | 418 | 144/418 | logistic regression | NI | 418 | 144/418 | * | * | * |  | C/U | 0.940 (0.888-0.991) | * | * | Yes | No |
| Hu, W | 2023 | 133 | 75/133 | logistic regression+ Comparative incorporation | split-sample V (7:3) | 94 | 53/94 | 39 | 22/39 | * |  | C/U/R | 0.88 (0.81–0.95) | 0.87 (0.75–0.99) | * | No | Yes |
| Huang, C | 2020 | 818 | 407/818 | LASSO | split-sample by admission | 512 | 257/512 | 306 | 150/306 | * |  | C/U | 0.765 (0.724–0.806) | 0.791 (0.740–0.842) | * | Yes | Yes |
| Huang, Y | 2023 | 344 | 240/344 | logistic regression | NI | 344 | 240/344 | * | * | * |  | C/B | 0.739 (0.681–0.797 | * | * | Yes | No |
| Jiang, L | 2023 | 211 | 88/211 | lasso+ logistic regression | split-sample V (7:3) | 148 | 59/148 | 63 | 29/63 | * |  | C/U | 0.700 (0.617–0.784) | 0.763 (0.650–0.877) | * | Yes | Yes |
| Li, J | 2022 | 729 | 313/719 | LASSO) regression and multivariate logistic regression | split-sample V (6:4) | 431 | 182/431 | 298 | 131/298 | * |  | C/U | 0.788(0.746–0.825) | 0.829(0.782–0.870) | * | Yes | Yes |
| Lin, P | 2021 | 423 | 185/423 | logistic regression | NI | 423 | 185/423 | * | * | * |  | C/U | 0.821 (0.768–0.875) | * | * | Yes | No |
| Liu, W | 2023 | 6650 | 2005/6650 | logistic regression | split-sample V (7:3) | 4247 | 1246/4247 | 1821 | 543/1821 | 582 | 216/582 | C/B | 0.706 | 0.702 | 0.734 | Yes | Yes |
| Pang, J | 2023 | 1394 | 676/1394 | lasso+ logistic regression | split-sample V (7:3) | 976 | 471/976 | 418 | 205/418 | * |  | C/U/B | 0.834 (0.809-0.860) | 0.803 (0.761-0.846) | * | Yes | Yes |
| Qiao, D | 2024 | 1392 | 853/1392 | logistic regression | split-sample by admission | 1009 | 607/1009 | 383 | 246/383 | * |  | C/U/B | 0.809 (0.784-0.833 | 0.755 (0.709–0.797 | * | Yes | Yes |
| Song, X | 2024 | 228 | 140/228 | logistic regression | split-sample by admission | 128 | 90/128 | 100 | 50/100 | * |  | C/U/B | 0.867 | 0.819 | * | Yes | Yes |
| Sun, F | 2021 | 1585+406 | 924/1991 | logistic regression | split-sample V (7:3) +EXV | 1094 | 483/1094 | 491 | 219/491 | 406 | 222/406 | C/U | 0.919 (0.902-0.935) | 0.921 (0.893-0.943) | 0.923 (0.893-0.947) | Yes | Yes |
| Wang, Z | 2022 | 2554 | 982/2554 | logistic regression | split-sample V (7:3) | 1787 | 703/1787 | 767 | 279/767 | * |  | C/U/B | 0.781 | 0.736 | * | Yes | Yes |
| Wei, L | 2024 | 114 | 62/114 | logistic regression | split-sample V (2:1) | 76 | 36/76 | 38 | 26/38 | * |  | C/U/R | 0.898 (0.830 ~ 0.965) | 0.853 (0.727 ~ 0.978) | * | Yes | Yes |
| Wen, Q | 2022 | 353+68 | 220/421 | logistic regression | split-sample by admission | 353 | 185/353 | 68 | 35/68 | * |  | C/U/R | * | 0.75(0.63-0.86) | * | Yes | Yes |
| Xue, J | 2023 | 129 | 61/129 | lasso+ logistic regression | split-sample V (7:3) | 90 | 41/90 | 39 | 20/39 | * |  | C/U/R | 0.934 (0.882 - 0.986) | * | * | Yes | Yes |
| Xue, T | 2021 | 379 | 162/379 | logistic regression | NI | 379 | 162/379 | * | * | * |  | C/U | 0.756(0.707-0.805 | * | * | Yes | No |
| Yang, Z | 2020 | 1438 | 618/1252 | logistic regression | split-sample by admission | 1252 | 618/1252 | 186 | * | * |  | C/U/B | 0.854 (0.843 - 0.867) | 0.825 (0.793 - 0.857) | * | Yes | Yes |
| Zeng, B | 2022 | 747 | 251/747 | logistic regression | bootstrap resampling | 747 | 251/747 | 374 | 135/374 | * |  | C/U | 0.703 | 0.701 | * | Yes | Yes |
| Zhang, H | 2020 | 214 | 100/214 | logistic regression | NI | 214 | 100/214 | * | * | * |  | C/U | 0.894 | * | * | Yes | No |
| Zhou, S. C | 2020 | 609+326 | 295/935 | LASSO+ logistic regression | split-sample by admission | 609 | 182/609 | 326 | 113/326 | * |  | C/U/B | 0.805 (0.746–0.864) | 0.766 (0.637 - 0.896) | * | Yes | Yes |
| -ng, Y | 2022 | 720 | 263/720 | logistic regression | MC | 300 | 104/300 | 143 | 47/143 | 144+133 | 112/277 | C/U/R | 0.875 (0.834–0.915) | 0.857 (0.785–0.929) | 0.880 (0.826–0.934)/0.870 (0.808–0.932) | Yes | Yes |
| Zhao, D | 2024 | 609 | 109/426 | logistic regression | split-sample V (7:3) | 426 | 109/426 | 183 | * | * |  | C/U | 0.782 (0.730-0.833) | 0.753 (0.648–0.858) | * | Yes | No |

## Table 2 Summarize the performance and calibration of the LLNM-group nomograms

| Author | year | Total sample enrolled | Total LNM rate | Statistical model | Validation method | Sample size (TRD) | LNM rate (TrD) | Sample size (INV) | LNM rate (InV) | Sample size (EXV) | LNM rate (ExV) | Feature categories | TRS | INT VAL | EXT VAL | Calibration plot | DCA |
| --- | --- | --- | --- | --- | --- | --- | --- | --- | --- | --- | --- | --- | --- | --- | --- | --- | --- |
| Chang, Q | 2022 | 1324 | 139/1324 | logistic regression | split-sample V (3:1) +EXV | 993 | 104/993 | 331 | 35/331 | 631 | * | C/U/B | 0.911 | 0.867 | 0.841 | Yes | Yes |
| Dong, L | 2023 | 1213 | 762/1213 | logistic regression | split-sample V (7:3) +EXV | 800 | 497/800 | 345 | 214/345 | 68 | 51/68 | C/R | 0.856 | 0.838 | 0.791 | Yes | Yes |
| Dou, Y | 2020 | 653 | 104/653 | logistic regression | NI | 460 | 72/460 | 193 | 32/193 | * |  | C/U/P | 0.835 (0.79 - 0.86) | 0.786 | * | No | Yes |
| Dou, Y | 2020 | 653 | 253/653 | logistic regression | NI | 460 | 186/460 | 193 | 71/193 | * |  | C/U/P | 0.855 (0.82-0.87) | 0.82 | * | No | Yes |
| Feng, J. W | 2022 | 528 | 115/528 | logistic regression | bootstrap resampling | 528 | 115/528 | bootstrap resampling |  | * |  | C/U/P | 0.864 | * | * | Yes | No |
| Feng, J. W | 2022 | 528 | 87/528 | logistic regression | bootstrap resampling | 528 | 87/528 | bootstrap resampling |  | * |  | C/U/P | 0.748 | * | * | Yes | No |
| Feng, J. W | 2022 | 1106 | 235/1106 | logistic regression | bootstrap resampling | 1106 | 235/1106 | bootstrap resampling |  | * |  | C/U/P | 0.758 | * | * | Yes | No |
| Heng, Y | 2020 | 434 | 142/434 | logistic regression | bootstrap resampling | 434 | 142/434 | bootstrap resamples |  | * |  | C/U | 0.761 (0.707–0.815) | 0.759 (0.745–0.773) | * | Yes | No |
| Liu, S | 2021 | 1198 | 177/1198 | logistic regression | NI | 1198 | 177/1198 | * |  | * |  | C/U/P | 0.714 | * | * | Yes | No |
| Liu, W | 2023 | 6650 | 813/6650 | logistic regression | split-sample V (7:3) | 4247 | 509/4247 | 1821 | 214/1821 | 582 | 90/582 | C/U/B | 0.818 | 0.791 | 0.762 | Yes | Yes |
| Ma, Y | 2023 | 336 | 139/336 | logistic regression | split-sample V (7:3) | 228 | 96/228 | 108 | 43/108 | * |  | C/U/P | 0.804 (0.745–0.863) | 0.731 (0.635–0.827) | * | Yes | Yes |
| Tong, Y | 2021 | 868 | 86/868 | logistic regression | NI | 600 | 55/600 | 286 | 31/286 | * |  | C/U/R | 0.946 ( 0.911-0.982) | 0.914； 0.842-0.987) | * | Yes | Yes |
| Tong, Y | 2022 | 720 | 76/720 | logistic regression | MC | 300 | 27/300 | 143 | 15/143 | 144+133 | 15/144+19/133 | C/U/R | 0.938 (0.887–0.989) | 0.905 (0.789–0.999) | 0.884 (0.785–0.984)/0.906 (0.841–0.971) | Yes | Yes |
| Wang, J | 2023 | 476 | 147/355 | LASSO+ logistic regression | split-sample V (3:1) | 355 | 147/355 | 121 | * | * |  | C/U/B | 0.795 | 0.792 | * | Yes | Yes |
| Zhao, L | 2022 | 873 | 128/873 | logistic regression | split-sample by admission | 702 | 106/702 | * |  | 171 | 22/171 | C/U/B | 0.860 (0.821-0.898) | * | 0.827 ( 0.724 ~ 0.929) | Yes | Yes |
| Zhu, J | 2023 | 2612 | 564/2612 | logistic regression | split-sample V (3:1) +EXV | 1732 | 374/1732 | 578 | 118/578 | 302 | 72/302 | C/U | 0.813 (0.790-0.835) | 0.815 (0.775-0.854) | 0.870 ( 0.822-0.917) | Yes | Yes |
| Zhuo, X | 2022 | 253 | 77/253 | logistic regression | split-sample by admission | 138 | 47/138 | 115 | 30/115 | * |  | C/U/R | 0.956 (0.925-0.986 | * | 0.915 (0.862-0.967 | Yes | No |
| Zou, Y | 2021 | 406+101 | 154/507 | logistic regression | split-sample V (7:3) | 280 | 90/280 | 126 | 38/126 | 101 | 26/101 | C/R | 0.899 (0.857- 0.931) | 0.905 (0.839- 0.950) | 0.912 (0.839- 0.959) | Yes | Yes |
| Gong, J | 2023 | 2166 | 438/2166 | logistic regression | NI | 2166 | 438/2166 | * |  | * |  | C/U/B | 0.702(0.667-0.736) | * | * | Yes | No |
| Huang, C | 2022 | 418 | 144/418 | logistic regression | NI | 418 | 144/418 | * |  | * |  | C/U/B | 0.940 (0.888-0.991) | * | * | Yes | No |

# Supplemental material 8—Pooled summary model power


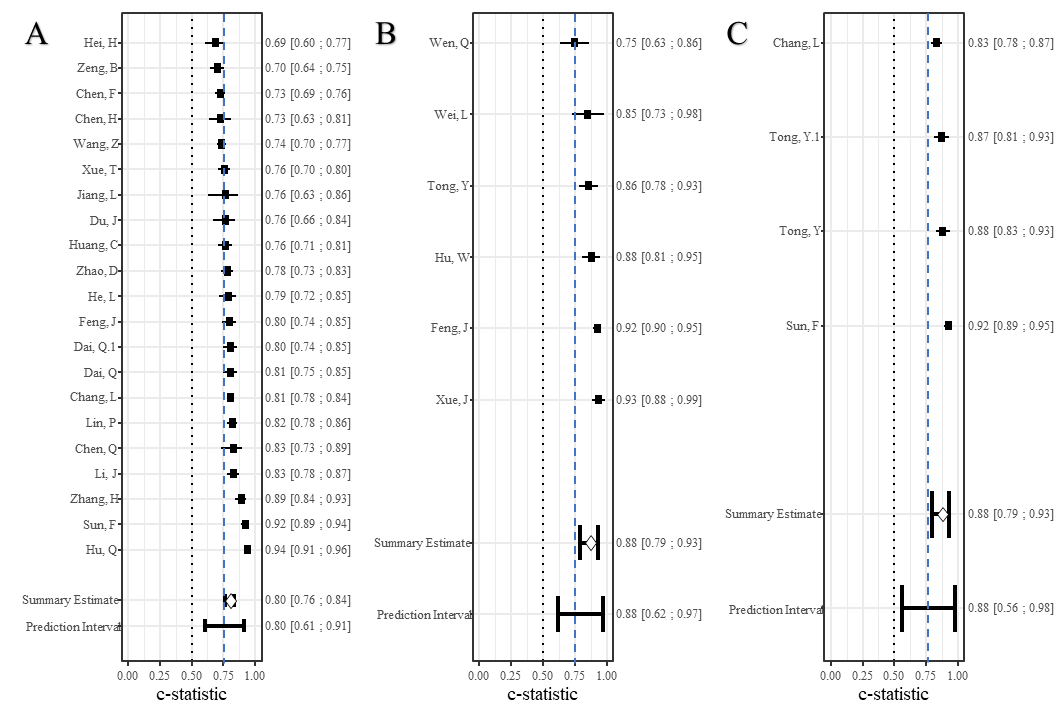


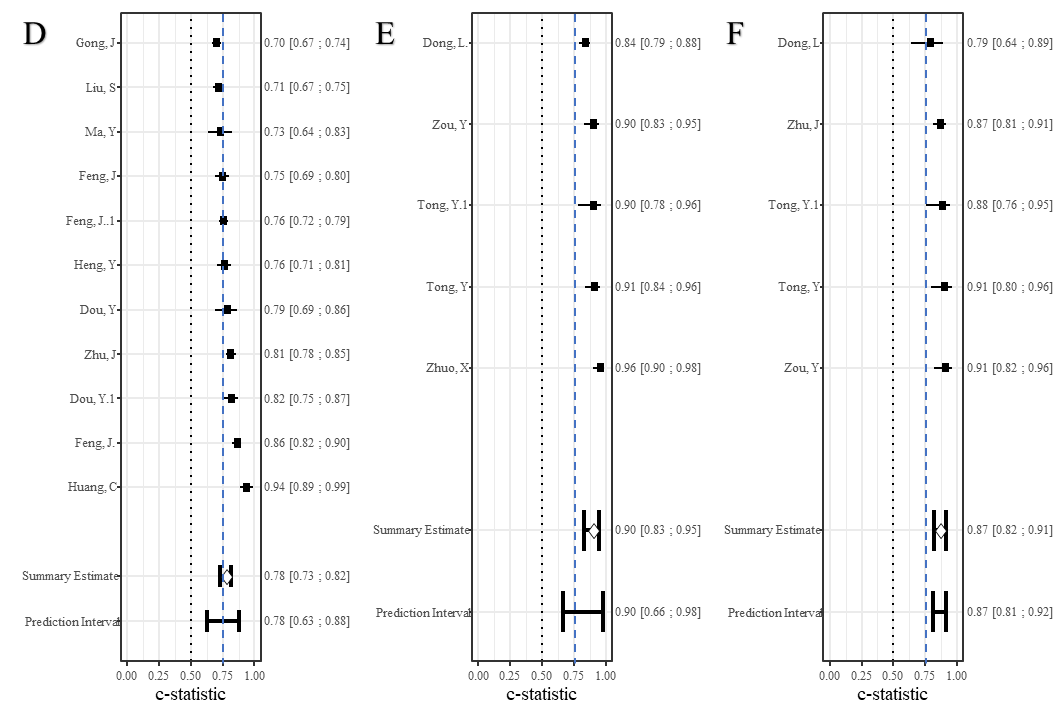


Figure 1 Subgroup meta-analysis of model pooled C-index: A, CLNM-US group; B, CLNM-RS group; C, CLNM external validation group; D, LLNM-US group; E, LLNM-RS group; F, LLNM external validation group.

## Table 1 Subgroup meta-analysis of model pooled C-index

| variables | N | OR(95%CI) | OR | LowerCI | UpperCI |
| --- | --- | --- | --- | --- | --- |
| Variables | Included models | AUC(95%CI) |  |  |  |
| CLNM |  |  |  |  |  |
| Ultrasonics | 21 | 0.80 (0.76 ; 0.84) | 0.8 | 0.76 | 0.84 |
| Radioics | 5 | 0.88 (0.79 ; 0.93) | 0.88 | 0.79 | 0.93 |
| LLNM |  |  |  |  |  |
| Ultrasonics | 11 | 0.78 (0.73 ; 0.82) | 0.78 | 0.73 | 0.82 |
| Radioics | 5 | 0.87 (0.82 ; 0.91) | 0.87 | 0.82 | 0.91 |

## Table 2 Subgroup meta-analysis of model pooled sensitivity

| variables | N | OR(95%CI) | OR | LowerCI | UpperCI |
| --- | --- | --- | --- | --- | --- |
| Variables | Included models | sensitivity(95%CI) |  |  |  |
| CLNM |  |  |  |  |  |
| Ultrasonics | 19 | 0.74(0.70-0.77) | 0.74 | 0.7 | 0.77 |
| Radioics | 6 | 0.84(0.76-0.90) | 0.84 | 0.76 | 0.9 |
| LLNM |  |  |  |  |  |
| Ultrasonics | 11 | 0.69(0.64-0.74) | 0.69 | 0.64 | 0.74 |
| Radioics | 5 | 0.83(0.80-0.86) | 0.83 | 0.8 | 0.86 |

## Table 3 Subgroup meta-analysis of model pooled specificity

| variables | N | OR(95%CI) | OR | LowerCI | UpperCI |
| --- | --- | --- | --- | --- | --- |
| Variables | Included models | specificity(95%CI) |  |  |  |
| CLNM |  |  |  |  |  |
| Ultrasonics | 19 | 0.76(0.71-0.80) | 0.76 | 0.71 | 0.8 |
| Radioics | 6 | 0.83(0.73-0.89) | 0.83 | 0.73 | 0.89 |
| LLNM |  |  |  |  |  |
| Ultrasonics | 11 | 0.75(0.70-0.80) | 0.75 | 0.7 | 0.8 |
| Radioics | 5 | 0.89(0.82-0.97) | 0.89 | 0.82 | 0.97 |
